# Creating a diagnostic assessment model for autism spectrum disorder by differentiating lexicogrammatical choices through machine learning

**DOI:** 10.1371/journal.pone.0311209

**Published:** 2024-09-27

**Authors:** Sumi Kato, Kazuaki Hanawa, Manabu Saito, Kazuhiko Nakamura

**Affiliations:** 1 Department of Neuropsychiatry, Graduate School of Medicine, Hirosaki University, Hirosaki, Japan; 2 Faculty of Management and Law, Aomori Chuo Gakuin University, Aomori, Japan; 3 Natural Language Processing Lab, Graduate School of Information Sciences, Tohoku University, Sendai, Japan; 4 Department of Clinical Psychological Science, Graduate School of Health Sciences, Hirosaki University, Hirosaki, Japan; University of Missouri Columbia, UNITED STATES OF AMERICA

## Abstract

This study explores the challenge of differentiating autism spectrum (AS) from non-AS conditions in adolescents and adults, particularly considering the heterogeneity of AS and the limitations of diagnostic tools like the ADOS-2. In response, we advocate a multidimensional approach and highlight lexicogrammatical analysis as a key component to improve diagnostic accuracy. From a corpus of spoken language we developed, interviews and story-recounting texts were extracted for 64 individuals diagnosed with AS and 71 non-AS individuals, all aged 14 and above. Utilizing machine learning techniques, we analyzed the lexicogrammatical choices in both interviews and story-recounting tasks. Our approach led to the formulation of two diagnostic models: the first based on annotated linguistic tags, and the second combining these tags with textual analysis. The combined model demonstrated high diagnostic effectiveness, achieving an accuracy of 80%, precision of 82%, sensitivity of 73%, and specificity of 87%. Notably, our analysis revealed that interview-based texts were more diagnostically effective than story-recounting texts. This underscores the altered social language use in individuals with AS, a crucial aspect in distinguishing AS from non-AS conditions. Our findings demonstrate that lexicogrammatical analysis is a promising addition to traditional AS diagnostic methods. This approach suggests the possibility of using natural language processing to detect distinctive linguistic patterns in AS, aiming to enhance diagnostic accuracy for differentiating AS from non-AS in adolescents and adults.

## Introduction

Autism spectrum (AS) is a neurodevelopmental condition characterized by persistent difficulties in social communication and interactions across various situations. Alongside this, individuals with AS exhibit repetitive and restricted patterns of behavior, activities, or interests [1]. The primary symptom revolves around challenges in social communication, primarily manifesting as pragmatic impairment (PI) [2, 3]. PI is characterized by specific difficulties in language comprehension and expression, especially at the pragmatic level, which pertains to the effective use of language in social contexts. This includes challenges in adapting language formality based on the situation, interpreting non-literal language (such as idioms, metaphors, irony, and sarcasm), and understanding the nuances of language that affect interpersonal interactions. It refers to struggles with these pragmatic aspects of language, rather than with the basic structural or grammatical components.

There is a widespread consensus among researchers in the clinical field that PI should be examined comprehensively, incorporating multiple factors like language, nonverbal aspects, and cognition. Previous studies have provided insights into the potential factors contributing to PI, indicating that it may arise from neurological, cognitive, symbolic, and/or sensorimotor dysfunctions [4–7]. Perkins [4] outlines four key domains of pragmatics, namely: (1) Semiotic: Encompasses language aspects (phonology, prosody, morphology, syntax, semantics, and discourse) and nonverbal elements (gestures, gaze, facial expressions, and posture). (2) Cognitive: Involves processes like inference, theory of mind, executive function, memory, along with emotions and attitudes. (3) Motor: Concerns physical aspects of communication (use of the vocal tract, hands, arms, face, eyes, and body). (4) Sensory: Focuses on hearing and vision for understanding and conveying information. Perkins’ classification prioritizes factors contributing to PI, highlighting cognitive dysfunction as the primary cause, with linguistic and sensorimotor factors deemed secondary.

Clinicians have observed individuals with AS who possess reasonably good language skills but struggle with effective communication. This has led them to recognize the vital role that cognitive functions, such as inferential reasoning, executive function, and memory, play in interpersonal interactions. Consequently, the clinical field has argued for a close association between cognition and PI [4]. As a result, neurology-based research, has become a major focus of studies of PI [5].

Previous studies regarding concrete linguistic phenomena of AS with cognitive perspective explored single grammatical areas such as modality [8–12], relative clauses [13, 14], and syntax [3, 15–19].

Investigating one such area, modality, Perkins [8, 9] and Nuyts and Roeck [10] conducted story-narrating experiments with AS children and reported limited understanding and use of epistemic modal expressions. Similarly, Kato [11] found individuals with AS were found to be less likely to utilize certain modal expressions, such as *probability* expressions (must, will, may, etc.) and *evidentiality* expressions (seems, looks like, likely, is said, according to, etc.). The study further revealed that the cognitive processes associated with *probability* and *evidentiality* are closely linked to the reasoning process. McDonald [20] argued that executive processes are primary among cognitive functions, with similarities between executive function and inference generation, noting that as impairment in the executive system increases, there is a corresponding increase in inferential reasoning difficulties. Autistic children struggle in situations where contextual information is not explicit and where they need to rely on general or social knowledge, as they excel more in deductive reasoning than inductive reasoning [21–23]. Such a reasoning pattern influences how AS individuals interpret and utilize the modal expression, *must* [21]. These two aspects of grammatical classification, *probability*, and *evidentiality* also point to a broader difficulty in utilizing context to derive meaning.

This ability to infer is related not only to the Executive Function Theory [24–27] but also to other cognitive theories such as the Empathizing-Systemizing Theory [28, 29] and the Weak Central Coherence Theory [30–33]. For Example, as previous studies on Central Coherence have shown [34–37], individuals with AS often face challenges not only in comprehending facial expressions and gaze direction in others but also in producing these non-verbal cues themselves. This difficulty arises from an inability to integrate information from various contexts and an impaired ability to prioritize social cues.

These investigations link the observed linguistic phenomena to explanations rooted in cognitive dysfunction. However, a limitation of these studies is that they concentrate solely on specific grammatical aspects, and as a result, the overall picture of the impairment remains uncharted. To gain a comprehensive understanding of PI as a whole, a systematic and comprehensive mapping is required, one that identifies and explores linguistic phenomena and instances of pragmatic disorder across various grammatical domains. However, such a comprehensive and systematic mapping of PI within the domain of linguistics has not been undertaken thus far.

Among the aids available to assist AS diagnosis, the Autism Diagnostic Observation Schedule Second Edition (ADOS-2) and the Autism Diagnostic Interview-Revised (ADI-R) are most commonly used. The ADOS-2 is a semi-structured AS diagnostic assessment aid that focuses on behavioral observations; the ADI-R is a standardized clinical caregiver interview that yields the developmental history and the current characteristics of patient functioning as perceived by the caregiver. The two tools are recommended to be used in combination; this approach has demonstrated the highest diagnostic validity [38].

The former, the ADOS-2, especially, is considered the *gold standard* diagnostic measurement [39, 40]. However, the results of some previous studies have led to questions regarding its versatility, particularly for adults [41, 42], for two main reasons. First, the ADOS-2 does not clearly differentiate AS from other neurodevelopmental conditions such as attention deficit hyperactivity disorder (ADHD), nor does it distinguish AS from psychiatric conditions like the negative symptoms of schizophrenia [43, 44] or psychiatric comorbidities (e.g., anxiety disorders, mood disorders, and avoidant personality disorders) [45–47]. Additionally, the inherent heterogeneity within the AS itself further complicates differential diagnosis. The overlapping symptoms [48, 49] across these conditions make diagnosis challenging. Second, factors such as masking behavior, compensation strategies [50, 51], and learned camouflaging [52] can conceal critical information about impairment, potentially leading to misdiagnosis. Additionally, although not directly related to ADOS-2 measurements, diagnoses of adults are often difficult because developmental reports from parents or caregivers are commonly absent. Patient *self-insight* is unreliable [30, 53]. Consequently, AS diagnosis requires input from multiple perspectives, and this study suggests that language analysis has the potential to serve as a supplementary diagnostic tool.

One effective approach to comprehensively map the PI of AS involves utilizing corpora of spoken language from individuals with AS. Despite limited research in this area, one notable corpus for English is Parish-Morris et al.’s [54], although it is not publicly accessible. Through this corpus, differences in speaking rate and inter-turn gaps between AS and non-AS individuals have been observed. Among the available open corpora, the Nadig AS English Corpus [55] contains transcripts of videotaped free play between AS children and their parents. This corpus provides a raw collection of simple linguistic data with no semantic information annotated. Similarly, the Asymmetries AS Corpus focuses on Dutch-speaking AS and Typically Developed individuals’ spoken language in its raw form [56].

**Fig 1:**
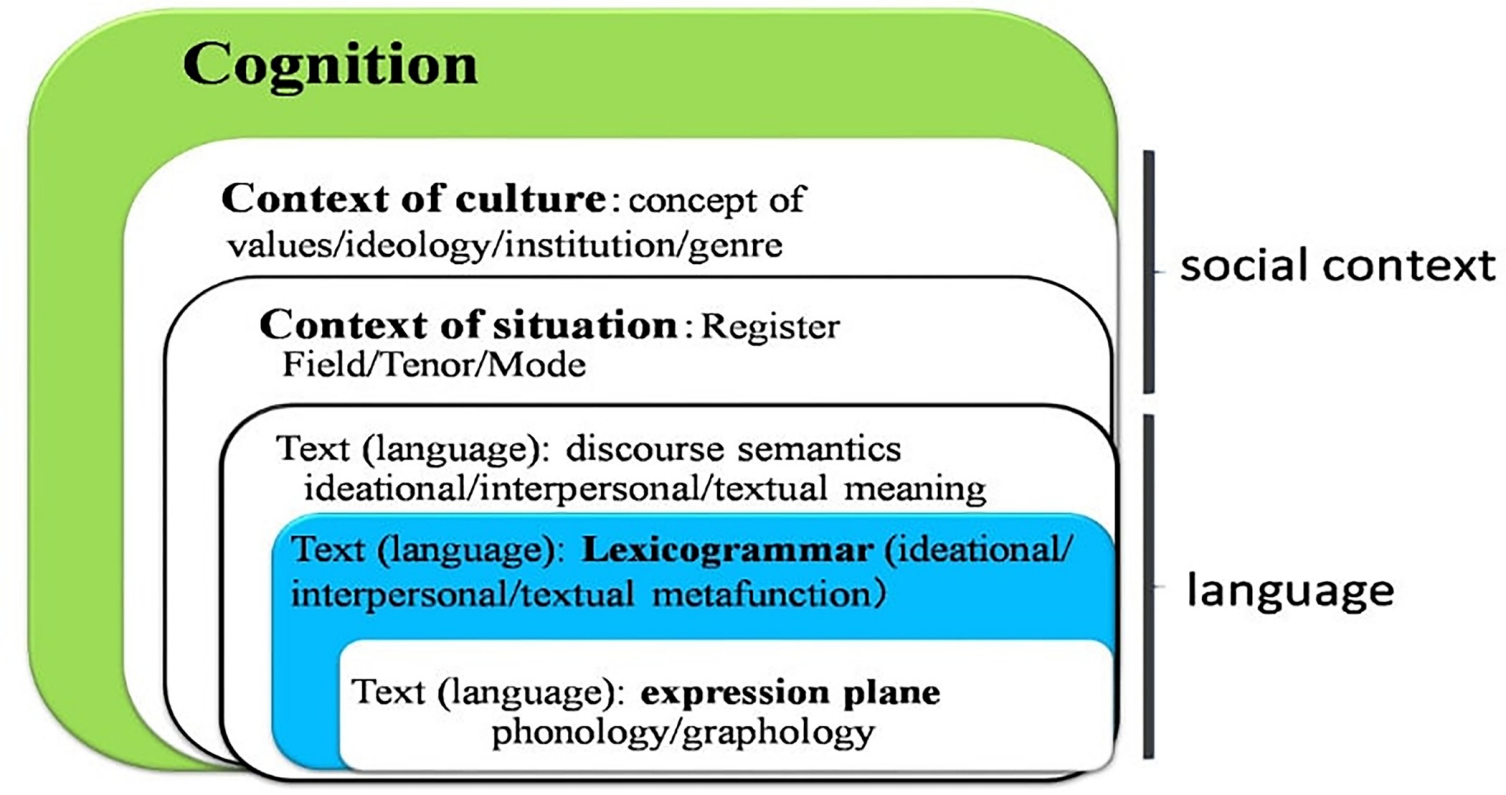
Position of cognition in language activities defined by SFL. (Adapted from Kato et al. [11]) This figure illustrates the SFL hierarchy enhanced by Kato’s cognition layer addition [11]. It demonstrates how culture and situation shape lexicogrammatical choices via Field, Tenor, and Mode, thereby impacting communication. The diagram underscores the importance of cognition in selecting suitable lexicogrammar for successful social interactions.

In studies of Japanese-speaking individuals with AS, Sakishita et al. [57] and Kato et al. [58] are notable for utilizing corpora specifically developed for their respective research. Sakishita’s corpus contains 17 types of annotations based on the publicly available Chiba 3 Party [59], with a primary focus on phonetic usage. The analysis involves examining statistics derived from these annotations and morpheme information, as well as investigating their relationship with the ADOS scores. On the other hand, Kato et al. [58] developed a comprehensive annotation scheme for analyzing syntax and lexicogrammar in spoken Japanese by individuals with and without AS. This scheme encompasses 159 annotation items derived from transcripts obtained during ADOS-2 administrations. The corpus in Kato et al. [58] was developed based on the theoretical framework of Systemic Functional Linguistics (SFL), which outlines interconnected layers of language activities as depicted in Fig 1.

The second stratum in Fig 1, encompasses *culture*, representing the collective social values and ideologies of a society associated with a specific language. The third stratum is defined by *situation*, encompassing *register* components that influence subordinate strata like *lexicogrammar* by shaping the lexicogrammatical decisions individuals make for meaningful communication. *Register* is composed of three elements: *Field*, *Tenor*, and *Mode*. *Field* addresses the questions "what is happening?" and "what is the topic?", covering ideational meanings. *Tenor* deals with the social and contextual roles of participants, representing interpersonal meanings, while *Mode* pertains to the communication channels during interactions, capturing textual meanings [60]; these elements are found in the fourth stratum. Interpersonal interactions are rooted in meaning choices, which are restricted to two specific contexts: *culture* in the second stratum and *situation* in the third. Ranking below, *discourse semantics* is expressed through *lexicogrammar*, with lexicogrammar subsequently articulated by *phonology/graphology*. A comprehensive grasp of both cultural and situational contexts empowers speakers to opt for fitting lexicogrammatical selections; in the absence of such understanding, they might choose inappropriately, leading to PI [58]. Variances in lexicogrammatical choices don’t always equate to PIs, with certain overlaps and disparities present. At the heart of PIs are socially unfitting lexicogrammatical selections.

SFL theory positions language purely as a social construct, sidestepping the cognitive aspect of meaning creation. Contrasting with SFL’s stance, Kato [11] posited that cognition should underpin social context, and consequently incorporated an additional layer dedicated to cognition at the outermost tier. A speaker’s proficiency in selecting socially suitable lexicogrammar hinges on their cognitive performance at this top layer. For instance, those with AS, stemming from cognitive anomalies like impaired executive function [61–64] or weak central coherence [32, 65–68], fail to accurately discern *culture* or *situation*. As a result, they resort to unsuitable lexicogrammatical selections [11].

In Kato et al.’s study, the corpus was specifically constructed to focus on the lexicogrammatical layer, the fifth layer. It is crucial within the framework of SFL to accord special attention to ’lexicogrammar’ due to its distinctive role in integrating vocabulary and grammar. Lexicogrammar is a term used in SFL to describe the interdependence of vocabulary and grammar. In SFL, grammar and vocabulary are not different strata, but are the two poles of a single continuum, properly called lexicogrammar. In other words, lexicogrammar is the grammar of the lexicon, where lexis (vocabulary) and grammar (syntax) combine into one. It is a level of linguistic structure where, in SFL, the grammar and the meaning of words are not separate systems, but are interdependent. Notably, there is no prior study that has presented a corpus with comprehensive annotation of the lexicogrammar of AS individuals’ spoken language. Kato et al.’s corpus [58] annotates syntax and lexicogrammar because PI in AS often manifests in skewed lexical choices, which are identified as lexical anomalies [69, 70]. Among the various challenges related to semantic choice in AS, lexical processing problems are the most frequently cited examples [69, 70].

Utilizing the aforementioned corpus, Kato et al. [58, 71] noted that Japanese individuals with AS showed a reduced tendency to use sentence-final particles (SFPs), specifically *ne* and *yo*, which facilitate calls-for-attention in interpersonal interactions. These particles, *ne* and *yo*, are seen as verbal indicators of joint attention. This observation points to potential issues with joint attention and weak central coherence in individuals with AS. Building on these observations, it’s noteworthy that children with typical development frequently use SFPs such as *ne* and *y*o by the ages of 1.5–2 years [72–74]. In contrast, studies indicate a marked reduction in the use of these SFPs among Japanese children with AS [75, 76]. Watamaki [76] associates the SFP *ne* with the development of empathy, proposing that its limited use in AS children might reflect social impairments in their language, a viewpoint supported by subsequent studies [77–79]. Furthermore, individuals with AS less frequently utilized evaluative lexis, which indicates dysfunctional joint attention and weak central coherence [80]. Thus, the results of previous studies suggested that language use could be diagnostic. However, no study has explored whether such an application is possible with respect to AS.

The objective of our research project is to develop a diagnostic tool for AS that utilizes natural language processing (NLP) technologies. This study marks the initial phase in proving the feasibility of such an instrument for evaluating lexicogrammatical choices. The hypothesis underlying this research is that the neurocognitive abnormalities associated with AS could be mirrored in language production, thereby creating specific AS-specific lexico-grammatical patterns that can be used to distinguish AS individuals from non-AS. Such patterns may be observed as variations from what is often considered *typical* speech in society, potentially suggesting linguistic behaviors that could be associated with neurocognitive differences in individuals with AS. Consequently, we propose that these abnormalities in neurocognitive function would manifest in language use; the lexicogrammatical choices made by those with AS would display distinctive trends that allow for diagnostic differentiation. These linguistic variances form the foundation for our differentiation algorithm.

To facilitate this, we utilized machine learning (ML) to analyze annotated corpus data. Previous studies have employed various machine learning techniques to enhance the diagnostic assessment of Autism Spectrum Disorder (ASD). For example, Schulte-Ruther et al. [81] used random forest models trained on ADOS item scores to predict ASD diagnoses amidst overlapping conditions, achieving high sensitivity and specificity. Similarly, Abbas et al. [82] combined questionnaire data with behavioral tagging from home videos, employing feature selection and engineering to improve early autism detection with increased accuracy. Levy et al. [83] explored sparse models using supervised learning methods on ADOS scores, achieving high ROC curve areas with minimal features, thereby offering a more interpretable and robust diagnostic approach. Duda et al. [84] validated a mobile autism risk assessment tool that demonstrated high sensitivity and specificity in clinical settings. Bone et al. [85] utilized support vector machines to enhance the effectiveness and efficiency of widely used ASD screening tools through ML-based algorithm fusion.

Despite these advancements, there remains a gap in the literature regarding the integration of lexicogrammar analysis within the ML framework for ASD diagnosis. None of the existing studies specifically incorporate the linguistic patterns of individuals with ASD as a criterion for classification. This study aims to bridge that gap by applying ML to the texts and annotations within the corpus to develop models capable of discriminating between ASD and non-ASD individuals, particularly through the analysis of lexicogrammar features.

## Methods

### Corpus as training database

#### Choice of corpus individuals

The database subjected to ML was the corpus of spoken language of AS individuals and non-AS individuals developed by Kato et al. [58]. We selected AS (*N* = 64, *M* = 18, *SD* = 3.48) and non-AS individuals (*N* = 71, *M* = 19, *SD* = 2.77) aged 14+ years, primarily between 14 to 20 year, post the critical period for language acquisition. This decision to select participants after the critical period is grounded in Lenneberg’s [86] and Newport’s [87] Critical Period Hypothesis (CPH), particularly referencing the Lenneberg hypothesis regarding the critical period for language acquisition and Newport’s hypothesis on biological constraints. The Lenneberg hypothesis posits that language learning intensifies over a distinct period during childhood and then diminishes with maturity, suggesting that language acquisition mechanisms align functionally with this critical period, typically concluding around puberty (ages 11 to 12). Newport’s ’less capacity leads to more learning’ hypothesis further elaborates that the primary learning mechanism decreases as cognitive processing capacities grow, leading to a competition that can limit further language acquisition.

While the CPH remains a topic of ongoing debate, there are supporting [88–95], opposing [96–98], and neutral [99–101] views on its validity. The consensus on the CPH does not overwhelmingly favor any single perspective. Acceptance of the hypothesis varies significantly depending on the specific language function being studied (e.g., syntax, phonology, morphology) and the context of language learning, whether it’s first-language or second-language acquisition. These differing perspectives are broadly summarized as follows:

1. **Pro-critical period perspective**:
  **Phonological and syntactic development**: Substantial evidence indicates age-related declines in the ability to achieve native-like pronunciation and syntactic proficiency, particularly noted in second-language acquisition.
  **First-language acquisition**: Research on individuals deprived of early language exposure shows profound deficits, supporting a critical period for language acquisition.
2. **Con-critical period perspective**:
  **Proficiency in late learners**: Some studies demonstrate that late learners can achieve high levels of linguistic competence, challenging the concept of a rigid critical period.
  **Neuroplasticity**: Neuroscientific studies highlight continued brain plasticity into adulthood, suggesting that language learning remains viable, though perhaps more challenging, beyond early years.
3. **Neutral/Mixed perspective**:
  **Variable acquisition timelines**: Research suggests that optimal periods for language learning exist but are not strict cutoffs; instead, they represent phases where the ease of acquisition decreases.
  **Role of external factors**: Motivation, exposure, and educational methods significantly impact language learning, often mitigating the disadvantages of starting later.

Given these perspectives, we adopted a pro-CPH stance to minimize variability from ongoing language development. By selecting participants aged 14 and above, we align with studies suggesting that post-puberty, language acquisition stabilizes, providing a consistent baseline for analyzing AS-specific linguistic features. This approach allows us to focus on understanding the consistent underutilization of lexico-grammatical resources by individuals with AS, rather than the variability in general language development.

We recognize that language learning and expression continue to evolve throughout life. However, our study targets specific lexico-grammatical resources potentially underutilized by individuals with AS, which are likely consistent across ages and less influenced by the natural aging process. This approach allows us to focus on analyzing linguistic characteristics associated with neurodevelopmental differences, providing a clearer understanding of AS-specific language use.

Individuals with AS underwent clinical diagnosis using the DSM-5 criteria, carried out by experienced clinicians who specialized in the diagnosis and treatment of neurodevelopmental disorders. The primary assessment tool for confirming the AS diagnosis was ADOS-2. Clinicians used several assessments alongside ADOS-2 for comprehensive evaluation (Table 1), including: (1) SRS-2 (social behavior and competency); (2) Intelligence Tests—WISC-IV for under 16 years, WAIS-III or IV for 16+ (cognitive functioning); (3) Vineland-II (adaptive behavior and functioning); (4) AQ (AS-associated characteristics); (5) PARS-TR (parent interviews for AS behaviors).

**Table 1:** Overview of demographic and clinical metrics in AS vs. non-AS populations.

Characteristic	AS Group (N = 64)	Non-AS Group (N = 71)	P-Value
Age (years)	18 ± 3.48	19 ± 2.77	0.00
Sex (M/F)	24/40	39/32	0.06
Education	N/A	College GPA range: 2.4–2.8	N/A
ADOS-2 Module 3	6.93 ± 1.38	2.75 ± 2.01	< 0.01
ADOS-2 Module 4	11.42 ± 3.55	4.22 ± 2.17	< 0.01
SRS-2 Total Score	85.53 ± 9.00	N/A	N/A
WISC-IV-IQ Full Scale IQ	81.22 ± 14.42	N/A	N/A
WAIS-III Full Scale IQ	91.33 ± 20.12	N/A	N/A
Vineland-II Composite	64.83 ± 22.53	N/A	N/A
AQ	36.64 ± 8.04	N/A	N/A
PARS-TR Preschoolers	13.54 ± 6.27	N/A	N/A
PARS-TR Adolescents & adults	24.30 ± 11.49	N/A	N/A

Measurements of ADOS-2 are based on both observation and interaction; an individual with suspected AS is assessed in terms of reciprocal social interaction, communication, and imagination in a semistructured setting. Coding of observed behaviors using scoring algorithms yields a diagnostic measure of autism symptoms. The scores are compared with AS cut-off scores. If an individual meets or exceeds the cut-offs for reciprocal social interaction, communication, and restricted and repetitive behaviors, that individual meets the criteria for a diagnosis of AS. ADOS-2 administrations were conducted by an administrator who established research reliability with the experience required to use ADOS-2 results in a research setting by Western Psychological Services.

**Fig 2:**
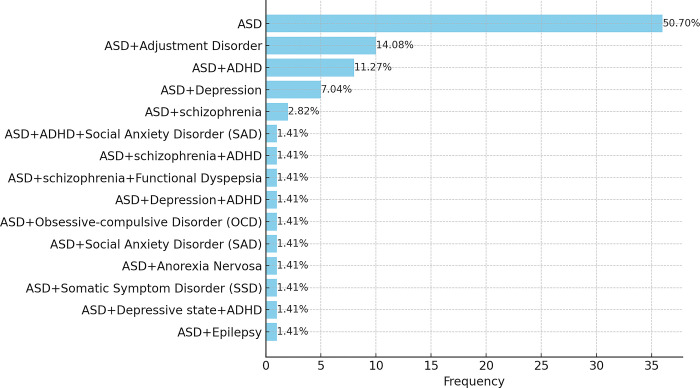
Distribution of AS and co-occurring conditions.

In our study, the AS cohort exhibited some comorbid conditions (Fig 2). It’s crucial to note that while AS is the primary diagnosis, the comorbidities are secondary to the core AS condition. The focus of this study is not to distinguish AS without co-occurring conditions from non-AS cases but to explore the identification of AS individuals, regardless of any comorbidities. We acknowledge the extensive research indicating that a substantial proportion of individuals with AS present with comorbidities [102–106]. Given that AS without co-occurring conditions may constitute only a small fraction of the spectrum, any diagnostic algorithm focusing solely on this subgroup would have limited clinical utility if it does not account for the broader AS population, which typically includes various comorbid conditions.

The non-AS group consisted of two different types of participants: (1) Participants who did not meet the criteria for a clinical diagnosis of any psychiatric disorder (*N* = 17). These participants were not included in the clinical group based on the final diagnosis. The determination of their primary diagnosis was conducted through a comprehensive diagnostic process, consistent with the methodology employed for the AS subject groups. ADOS-2 scores indicated no AS signs: Module 3 average was 3.17 and Module 4 was 4.00 for communication and social interaction, confirming non-spectrum status according to ADOS-2 criteria. For ADOS-2, scores of 6 or below in Module 3 and a combined score of 7 or below in communication and social interaction for Module 4 are considered non-spectrum. This population did not include comorbidities of neurodevelopmental disorders. (2) Participants, primarily college students (N = 54), who were recruited and assessed as non-AS, indicative of not exhibiting AS characteristics according to the ADOS-2 assessment by a research reliability-established administrator. Their ADOS-2 scores confirmed the absence of AS signs: a mean of 2.01 in Module 3 and 4.27 in Module 4 for communication and social interaction total, indicative of non-spectrum status as per the established scoring criteria of the ADOS-2. While IQ testing was not part of our methodology, we selected individuals with a GPA range of 2.4 to 2.8, a range statistically representative of the average for Japanese college students [107–109], who are also recognized for their proficiency in social activities both within and beyond the academic setting. This selection approach, focused on social adaptability and functionality, was key to our study’s aim of examining social capabilities, particularly in contrast to the challenges frequently associated with AS. Additionally, there are some high school students and adults who were also given the ADOS-2 test and are recognized as proficient in social activities.

#### Ethics statement

This research was conducted from September 2, 2013, to October 5, 2020, in strict adherence to the ethical standards outlined in the Declaration of Helsinki. The study protocol was approved by the Hirosaki University Committee on Medical Ethics under IRB number 2013–142, with subsequent updates leading to the current approval under 2018–168, Previous Number: 2015–055. To safeguard personal data, we followed the committee’s information security guidelines closely. Participants aged 20 and above provided written consent, and for those 19 and under, we obtained written consent from both the participants and their parents or guardians. We used alphanumeric characters to anonymize participants and removed any identifiable utterances from the transcripts to protect their privacy. The recruitment and the retrospective analysis of diagnostic data occurred simultaneously, with the period spanning from September 2, 2013, to October 5, 2020. This dual approach involved both prospective recruitment of participants and the retrospective examination of their diagnostic data, treated with the same ethical rigor and adherence to privacy standards as outlined above.

#### The texts

The ADOS-2 uses five modules arranged according to language level and participant age. Modules 3 and 4 are used to elicit interview responses and story recounting, primarily assessing adolescents and adults with fluent speech. These modules are designed for verbally fluent individuals, where verbal fluency is defined as language development at or above the level of a typical 4-year-old child’s expressive skills. This includes the ability to produce various sentence types and grammatical forms, describe events beyond the immediate context, and use logical connectors like *but* or *though*, although occasional grammatical errors may occur [110]. Module 3 is typically suited for verbally fluent children and adolescents, incorporating tasks like playing with action figure-type toys, seen as age-appropriate for this group. Conversely, Module 4 is tailored for older adolescents and adults and does not include the action figure play task, although the other tasks remain largely the same.

Participants suspected of having AS were audiotaped while performing six to eight tasks in Modules 3 and 4. These audiotapes were then manually transcribed to ensure high accuracy. Transcripts of these tasks were annotated and subsequently stored in our existing corpus. From this corpus, texts corresponding to targeted age groups were selected. We chose two different types of texts from the corpus: interview texts and participant-narrated narrative texts from ’Tuesday’ by Wiesner [111], a wordless picture book:

1. **Interview texts**: The interview questions in both Modules are designed to assess participant insights into personal difficulties, sense of responsibility, sense of social situations, and understanding of relationships (e.g., friendship, marriage, and family ties) (S1 File). Some questions explore imaginary-world creations, an objective description of self, and a description of one’s own emotions. According to the protocol, the examiner takes a conversational tone, avoiding a question-and-answer approach, and tries to further develop interaction by commenting on what the participant says (i.e., by showing interest). We used all specified questions in the ADOS-2 manual to ensure consistency in the assessment process.
2. **Story-recounting texts:** The story-recounting task assesses the participant’s ability to recount a wordless picture book; the participant also spontaneously describes the supposed emotional states of characters, such as how they are feeling.
  Interviews and story-recounting tasks tap into different cognitive and linguistic processes. Interviews, being dialogic, require immediate interactive communication that deeply engages social and pragmatic skills. In contrast, story-recounting, though monologic, also involves considering the listener’s perspective, making the narrative comprehensible and engaging in a more indirect way. Both types serve to explore social cognition and pragmatic abilities in AS, with interviews demanding direct social interaction and story-recounting engaging social cognition through narrative construction and anticipation of the listener’s needs.

In compiling our corpus, we included both the spoken texts of participants and the interviewer’s questions. However, our analytical focus was exclusively on the participants’ responses. The study examines the lexicogrammatical choices of individuals with AS in reciprocal social interactions, concentrating on their language use rather than the dynamics of interaction. Our approach evaluates patterns of lexicogrammar selection from participants’ responses, comprehensively analyzing their language use throughout the task. We assessed their entire spoken output during the evaluation, without setting specific requirements for text length, speaking duration, or word count.

#### Annotation scheme of the corpus

When developing the annotation scheme, Kato et al. [58] constructed four system networks (lexicogrammatical option systems from which speakers make choices) using the theoretical framework of SFL. Language choice is a central organizing concept of SFL. Individuals utilize different expressions based on various factors, such as the person they are addressing, the social setting, and other contextual elements. Consequently, when constructing a clause to convey a speaker’s intended meaning, there are multiple options available. The speaker, at the time of utterance, instantaneously makes choices through resource-selection mapping for each component of the clause. SFL defines this resource-selection mapping as the system network, encompassing all potential lexicogrammars that a speaker can select during linguistic interaction. Language, in this framework, is viewed as a meaning-making system where speakers draw upon resources from the system network as they engage in social activities [112]. In essence, the system network represents the vast array of linguistic choices available to speakers, and they actively choose from this network to express their intentions effectively and contextually during communication.

To better illustrate how individuals with AS make lexicogrammatical choices from a range of options within the system network, we examined their responses to the interview question ’How about feeling relaxed or content? What kinds of things make you feel that way?’ This involved analyzing three distinct lexicogrammatical choices, highlighting their decision-making process in language use.

Example 1 (Declarative): I find solace in nature, which helps me relax.

Example 2 (Interrogative with modalization:ability, *can*): Can spending time in nature help you feel relaxed?

Example 3 (Declarative with modulation:obligation, *must*): You must spend some time in nature to unwind and find relaxation.

**Fig 3:**
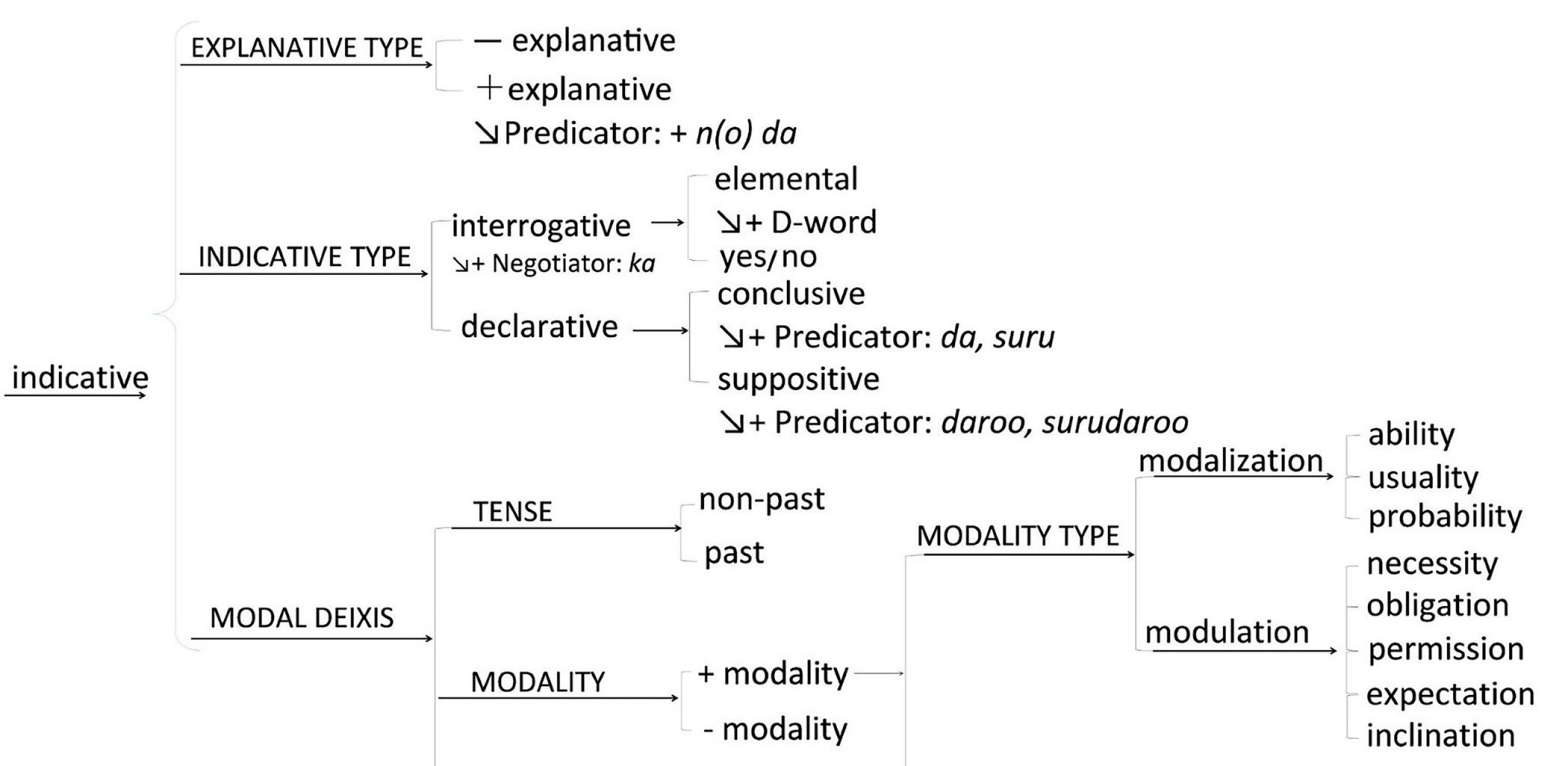
Indicative sentence type of the system network. This figure presents a close-up of the segment highlighted by the red circle in the MOOD system (S1 Fig 1 in S1 File for context). It illustrates the progression of delicacy in mood selection choices, moving from left to right across the network.

These examples exhibit how participants express similar ideas using different lexicogrammatical choices. To analyze these sentences, we refer to the mood selection network shown in Fig 3, which is an enlargement of the red-circled part of the MOOD system (S1 Fig 1 in S1 File). Delicacy increases from left to right on the mood selection network.

Indicative has three choices, EXPLANATIVE TYPE, INDICATIVE TYPE, and MODAL DEIXIS. Example 1 takes a declarative form from the INDICATIVE TYPE without taking EXPLANATIVE nor MODAL DEIXIS. The participant articulates their feelings through a definitive declarative statement, presenting a clear and unequivocal assertion. This sentence structure leaves little room for interpretation or doubt regarding their experience. The use of straightforward, declarative language conveys a strong, assertive claim about what the speaker intends to say.

On the other hand, in Example 2, the participant navigates the mood selection network from a general interrogative form towards a more specific yes/no question, which seeks to determine the likelihood of relaxation in nature. This progression is illustrated by the use of MODALITY, specifically modalization of ability (*can*), indicating a move from left to right on the network towards increased delicacy in lexicogrammatical choices. The intention could be seen as seeking information or confirmation from the listener. The participant appears to inquire whether spending time in nature has the potential to induce relaxation, aiming for a direct response. Additionally, this question might also serve a rhetorical or reflective function, potentially implying a broader, commonly held belief that nature inherently provides relaxation, rather than solely seeking the listener’s personal viewpoint.

In Example 3, the speaker employs the MODALITY TYPE alongside a declarative mood, specifically using modulation in the form of obligation (*must*). This choice underlines the necessity of engaging with nature for relaxation. By integrating modulation, obligation, the speaker asserts the essentiality of this action and aims to convince the listener of its importance for achieving relaxation. This linguistic strategy indicates a deliberate move to higher delicacy in the system network, reflecting the speaker’s persuasive intent.

These examples aim to emphasize the diverse language choices made by participant in response to similar prompts, highlighting the central role of linguistic choice in their communication.

**Fig 4:**
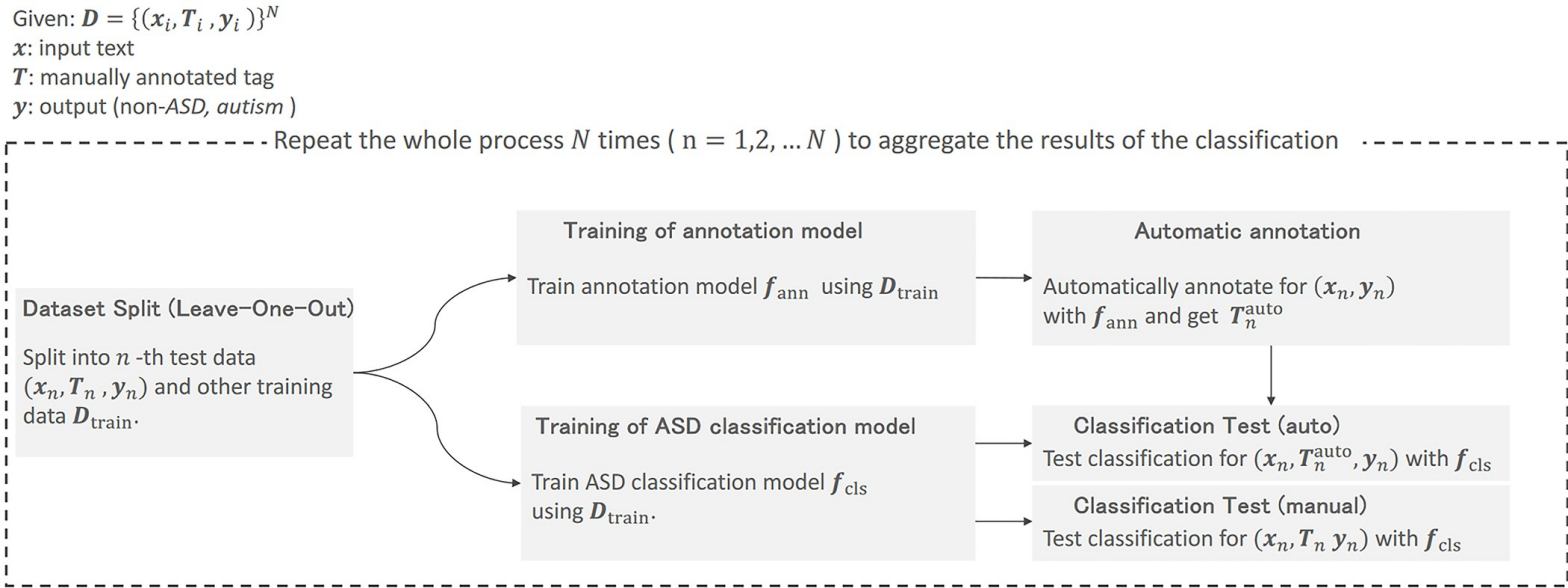
Procedure for automatic AS differentiation experiments using leave-one-out cross validation (LOOCV).

The system network is divided into several parts based on the SFL lexicogrammatical classification. The Japanese system networks that Kato et al. [58] constructed are (i) MOOD (S1 Fig 1 in S1 File), (ii) APPRAISAL (S1 Fig 2 in S1 File), (iii) TRANSITIVITY (S1 Fig 3 in S1 File), and (iv) LOGICAL (S1 Fig 4 in S1 File). Kato et al. [58] charted all possible lexicogrammatical resources within these four systems, creating a network of interconnected options. The annotation scheme was formulated based on these networks. However, annotation does not encompass all the lexicogrammatical resources within the network, but only includes resources located in the green sections as shown in S1 Figs 1–4 in S1 File.

In light of the neurocognitive characteristics often observed in individuals with AS, our objective was to identify lexicogrammatical selections characterized by their differential usage—both those less and more frequently employed by this group compared to non-AS individuals, drawing upon the studies mentioned previously. Each identified lexicogrammar is thought to necessitate certain cognitive abilities for its effective utilization, similar to how joint attention may be required for the use of SFPs as discussed earlier.

Table 2 shows the tag set scheme and the lexicogrammatical functions used by Kato et al. [58] to annotate the scheme constructs. Each of the 15 headings has distinct subcategories; there are 147 different tag types (Table 2).

**Table 2:** Tag types and linguistic functions.

Lexicogrammar headings	Linguistic functions	Tag types	No. of tag types
**Ideational metafunction**			
1. Process type	The mental image of reality is constructed by the TRANSITIVITY (clause component) of a clause. All individuals create a representation of reality. Experiential worlds are defined using 10 types of process verbs, yielding information about how, when speaking, an individual creates a representation of reality.	1.Material-doing 2.Materisal-happen 3.Mental-cognition 4.Mental-affect 5.Mental-perception 6.Relational-attribute 7.Relational-identity 8.Behavioral 9.Verbal 10.Existential	10
2. Ergativity	This measures causation or instigation. In an ergative analysis, the participant that causes an event is the agent. Ergativity reveals whether a speaker interprets events and reality from the causal viewpoint of agency (effective) or becoming (i.e., a perspective lacking agency; a *middle*).	1.effective 2.middle	2
3. Transitivity	A property that yields clues regarding the perspective (active or passive) from which the speaker interprets events and reality.	voice (1.passive/active 2.causative)	2
4. Clause complexes	The Japanese sentence type (of 22 types) chosen. This reveals syntactic ability and any cognitive tendency or deficiency.	1.Parallel clause 2.Te-form/Conjunctive clause-parallel/contrast 3.Te-form/Conjunctive clause- forerunner 4.Te-form/Conjunctive clause-sequence of actions 5.Te-form/Conjunctive clause-cause/ reason 6.Te-form/Conjunctive clause-adversative connective 7.Te-form/Conjunctive clause-resultative condition 8.Te-form/Conjunctive clause-attendant circumstance 9.Conditional clause-resultative condition 10.Conditional clause-converse condition-converse condition 11.Conditional clause-converse condition-adversative connective 12.Conditional clause-cause/ reason 13.Purpose clause 14.Time clause-temporal anteroposterior relation 15.Time clause-simultaneous actions 16.Time clause-others 17.Manner clause 18.Reported clause 19.Interrogative clause 20.Noun clause 21.Adnominal clause 22.Cordinate clause	22
5. Logico- semantic relation	Logical clause linkages revealing syntactic ability, discourse strategy, and any cognitive tendency or deficiency.	1.Expansion-elaboration-expository 2.Expansion-elaboration-exemplifying 3.Expansion-elaboration-clarifying 4.Expansion-extension-additive 5.Expansion-extension-alternative 6.Expansion-enhancement-temporal 7.Expansion-enhancement-spatial 8.Expansion-enhancement-manner 9.Expansion-enhancement-cause-conditional 10.Projection-quote 11.Projection-report 12.Projection-idea 13.Projection-embedding	13
6. Auxiliary verbs	**Stative:** Verbs describing the state of a subject rather than an action, reflecting the perspective of a speaker on an ongoing phenomenon.	stative: (19 categories) compound: (1 category)	20
	**Compound:** Verbs created by adding one verb to the stem of another; use of these verbs reflects the morphological skill of a speaker.		
**Interpersonal metafunction**			
7. Modality	In SFL, modality refers to an area of meaning that lies between yes and no; this constitutes the intermediate space between positive and negative polarity, categorized as either modalization (epistemic modality) and modulation.	1.Ability 2.Probability 3.Usuality 4.Necessity 5.Obligation 6.Permission 7.Expectation 8.Inclination	8
8. Appraisal- attitude	The semantic resource used to negotiate emotional reactions, judge behavior, and value things. Attitude is divided into three domains: affect, judgment, and appreciation. Affect is used to interpret emotional responses (including fear, loathing, sadness, and happiness); judgment is used for moral evaluation of behavior (including ethical, brave, and deceptive); and appreciation is used to interpret the esthetic qualities of semiotic phrases/processes and natural phenomena (including remarkable, desirable, elegant, harmonious, and innovative). This lexicogrammar reveals the speaker’s value system.	1.AFFECT-inclination 2.AFFECT-emotion 3.AFFECT- security 4.AFFECT-satisfaction 5.JUDGEMENT-capacity 6. JUDGEMENT-reliability 7.JUDGEMENT-veracity 8.JUDGEMENT-propriety 9.JUDGEMENT-propencity 10.APPRECIATION-reaction 11.APPRECIATION-composition 12.APPRECIATION-phase-time 13.APPRECIATION-phase-extent 14.APPRECIATION-phase-degree 15.APPRECIATION-phase-space 16.APPRECIATION-phase-distance 17.APPRECIATION-phase-mass 18.APPRECIATION-social evaluation	18
9. Appraisal- graduation	This is one of the three categories that make up Appraisal, along with Appraisal-attitude, which focuses on gradability. (i.e., adjustment of the extent of evaluation).	1.FORCE-intensification 2.FORCE-quantification 3.FOCUS-sharpening 4.FOCUS-softening	4
10. Negotiating particle	A lexis that adds various negotiatory values to a clause, implying the attitudinal stance of a speaker toward a proposition or proposal; this lexis is associated with a call for attention and indicates the territory of the information involved.	sentence-ending particles; 1.*kana* 2.*kane* 3.*sa* 4.*ne* 5.*yo* 6.*yona* 7.*yone*: Particle- 8.*kane* 9.*sa* 10.*ne* 11.*yo*- at places other than the end of the sentence: other- 12.*ne*	12
11. Explanative mood	An optional lexicogrammar often added to other mood types such as declarative and interrogative, implying a variety of meanings. It constitutes a cause, reason, motivation, source, and/or grounds for judgment that suggest a causal relationship between the explained and the explainer.	1.Explanative mood 2.Explanative mood-*ka* 3.Explanative mood-*kana* 4.Explanative mood-*kane* 5.Explanative mood-*kedo* 6.Explanative mood-other 7.Explanative mood-*na* 8.Explanative mood-*ne* 9.Explanative mood-*yo* 10.Explanative mood-*yone* 11.Explanative mood-*yona* 12.Explanative mood-*monoda*	12
12. Evidentiality	This lexicogrammar describes how a speaker judges the validity of a proposition. Three types of evidence are used. *Appearance* refers to how the information is likely to appear or eventually occur; *hearsay* refers to how it will be known whether the event occurs; and *reasoning* refers to the reason the judgment is made or how the event is known to happen.	1.appearance 2.hearsay 3.reasoning	3
13. Optative mood	A *desire* or *urge* to do something that the speaker considers desirable.	lexis to express desire to do something	1
14. Auxiliary verbs, benefactive	Verbs used when two parties converse; one party is doing something that benefits the other, and the other party is the recipient of that benefit. Such verbs indicate whether the speaker positions the other party inside or outside.	Benefactive: (10 categories)	10
15. Onomatopoeia	Imitative and mimetic words used to express manner, quality, or an exclamation.	1.imitative word 2.imitative mimetic word	2
16. Filler	A time-filler: a meaningless sound, word, or phrase used in social settings when an individual is aware that a listener is present.	Filler words-1.*maa* 2.*nanka* 3.*ano* 4.*unto* 5.*eeto* 6.*sono* 7.*kono* 8.*kou*	8
			Total	147

### Diagnostic differentiation by ML

#### Overview and rationale for machine learning approaches

In this study, we chose to explore both a linear model (logistic regression) and deep neural network (DNN) models to differentiate between AS and non-AS individuals, with a focus on the trade-off between interpretability and performance. Logistic regression was selected due to its simplicity and clarity, allowing us to examine the relationship between specific linguistic tags and the likelihood of AS. This high interpretability is crucial when the goal is to understand the significance of each linguistic feature in the classification process.

Although logistic regression was the sole linear model used, it was chosen deliberately for its well-established effectiveness and simplicity in binary classification tasks, which aligns with our objective of exploring interpretable models. Other linear models, such as linear support vector machines (SVMs), were considered but not included in this study due to their more complex implementation and the specific focus on the interpretability of the relationship between features and the outcome. The choice of logistic regression ensures that our findings remain directly interpretable, which is vital for the analysis of linguistic features.

To complement this, we also employed DNN models, which, while less interpretable, offer the potential for higher accuracy by capturing more complex patterns within the data. We proposed four models: a linear model using only tags, a DNN model using only tags, a DNN model using only text, and a DNN model that incorporates both tags and text.

It is important to clarify that the primary aim of this study was not to determine the best possible model or to establish the upper bounds of classification accuracy. Instead, the chosen models were intended to serve as tools to explore specific research questions related to linguistic feature analysis in the context of AS classification. The focus was on providing insights into the relationships between features and outcomes rather than exhaustively comparing model performance.

#### Input

Each text uttered during the interview and story-recounting phase served as the input, devoid of any annotations, treated by the machine as simple sequences of words. Therefore, each input was defined as ***x*** = (*w*_1_, *w*_2_,…,*w*_*L*_), where *w*_*i*_ denotes words and *L* signifies the count of these words. ML necessitates annotations; text annotated manually is generally preferable owing to its greater accuracy. However, this process is time-intensive, costly, and requires expertise, rendering manual annotation impractical in clinical environments. Consequently, we employed automatic annotation. The obtained F1 score, precision, and recall were 0.88, 0.89, and 0.87 respectively. These results are considered reliable enough to be used for distinguishing between groups.

#### Output

The output, denoted as ***y***, is classified as either AS or non-AS. This classifier is expressed as ***y*** = ***f***(***x***), and our objective was to identify an ***f*** that would predict ***y*** with the highest possible accuracy.

#### Experimental procedure

The data used were sets of quadruplets (***x***, ***T***^manual^, ***y***), where ***T***^manual^ denotes a manually annotated tag. Therefore, the dataset ***D*** is represented as $D={\left\{\left(x_{i},T_{i}^{manual},y_{i}\right)\right\}}^{N}$. Considering the small size of our dataset, we performed leave-one-out cross validation (LOOCV), where each sample serves as a test sample once while the remaining samples are used for training. This method involved 64 samples in the AS group and 71 in the non-AS group. For each iteration, the model was trained on n−1 samples and tested on 1 sample, as illustrated in Fig 4. Initially, we classified the n-th data as test data $\left(x_{n},T_{n}^{manual},y_{n}\right)$ and the remaining data as training data ***D***_train_. Following this, we trained the tag annotation model using ***D***_train_. The trained tag annotation model was then used to automatically annotate ***x***_***n***_, resulting in $T_{n}^{auto}$. This approach served as a replacement for manual annotation of ***T***^manual^ in real cases; we also present the results of manual annotation ***T***^manual^ for comparative purposes. Next, we trained the classification model using ***D***_train_. We then classified $\left(x_{n},T_{n}^{auto},y_{n}\right)$ and $\left(x_{n},T_{n}^{manual},y_{n}\right)$ using the trained model. Lastly, we consolidated the results of all tests (*n* = 1,2,…,*N*) and computed the accuracy, precision, recall, and specificity.

#### AS differentiation model

The study proposes three models for differentiating AS: a linear model using only tags, a DNN model also relying solely on tags, and a DNN model that incorporates both tags and text.

The study sets out to make two key comparisons. The first comparison evaluates a linear model that utilizes only linguistic tags against a DNN model that also relies solely on tags. This aims to explore the trade-off between interpretability and performance by juxtaposing the linear model’s high interpretability but lower efficacy with the DNN model’s superior performance but reduced clarity. The second comparison assesses the effectiveness of DNN models when inputs are limited to tags versus when both text and tags are incorporated. Given the potential of DNN models to extract comprehensive information from their inputs, this analysis seeks to assess the extent to which tags alone can encapsulate the informative essence of the original sentences for AS classification.

**(1) AS Differentiation Using Tags**

Given that the frequency of annotated tags differs between AS and non-AS individuals (S2 Tables 1 and 2 in S2 File), we hypothesize that AS differentiation can be effective through the use of tags. As each clinical input is a sequence of words devoid of tags, automatic annotation is essential. We approached such annotation as a sequence labeling problem, which we resolved using Bidirectional Long-Short Term Memory (Bi-LSTM), a type of deep neural network (DNN) within the realm of Machine Learning.

In formal terms, we computed a sequence ***T***^auto^ from the input ***x***, with ***T***^auto^ being a set of *C* types of tags. Here, ***T***^auto^ is an *L*×*C* m atrix where each row signifies a tagging category, and each column represents a word. Since all texts in the corpus have been manually annotated, we employed these texts for training differentiation models to ensure accuracy. Although manual annotation is not feasible in clinical settings, we used manually annotated texts to compare the accuracies of two methods, one based on a linear model and the other on a DNN.

For the linear model, we employed logistic regression. This method is transparent and hence, interpretable; it allowed us to identify tags that impacted the outputs and quantify these effects. Generally, differentiation by a linear model is often less accurate than by a DNN-based model, with the former exhibiting less precision [113, 114]. Nevertheless, in the medical context, interpretability is crucial because we are dealing with human lives. Therefore, it is not possible to make an unconditional judgment of which model is *better*. In the linear model, only tag frequencies were used as inputs, disregarding the order of tag occurrences. Thus, the input was ***x***_tag_ = (*t*_1_, *t*_2_,…,*t*_*C*_), where *t*_*i*_ is the frequency of the *i*-th tag divided by the total number of words.

**Fig 5:**
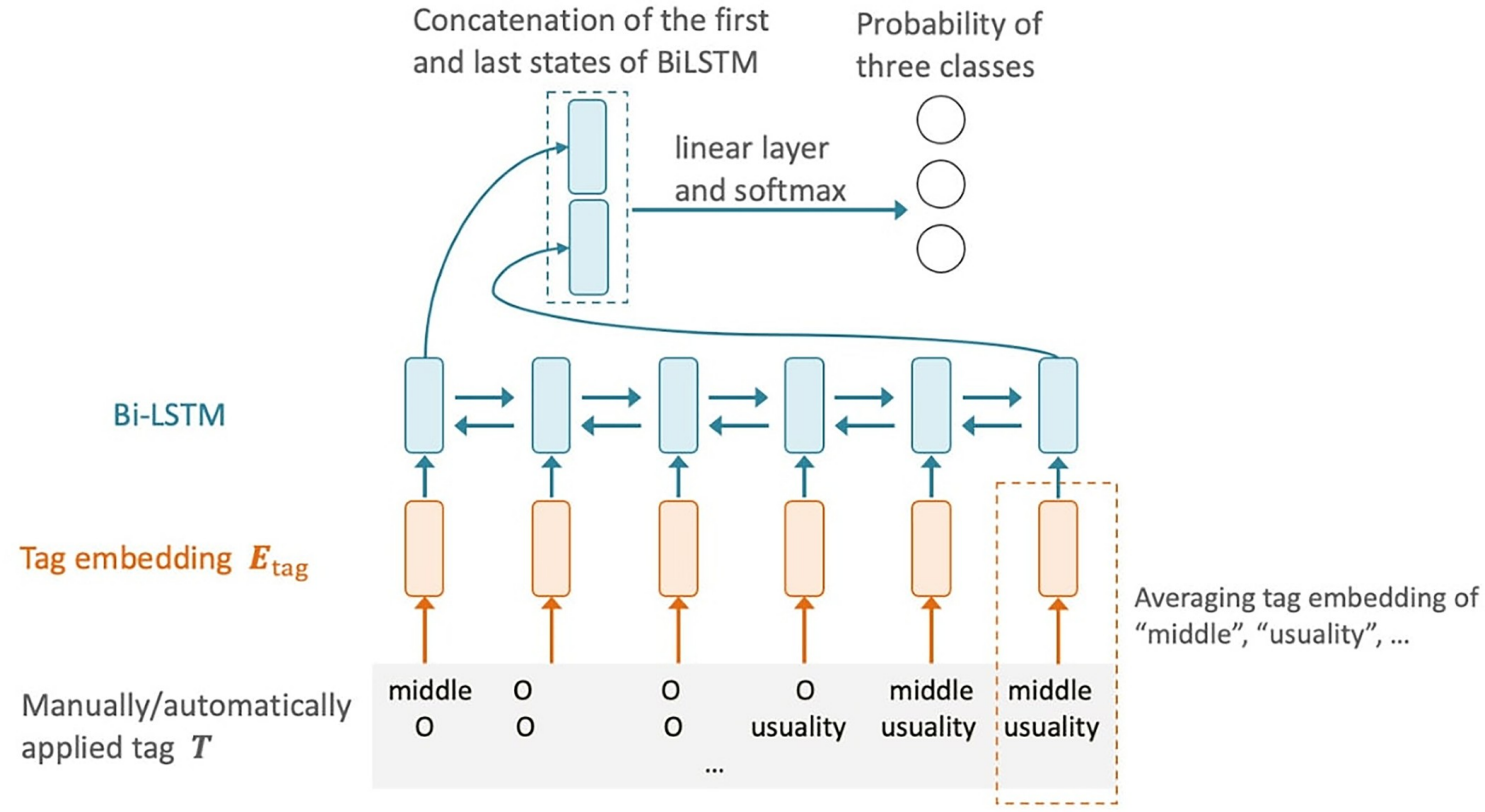
Bi-LSTM-based classification model using tags. The term *Middle* signifies a type of verb that lacks agency from a perspective, while *Usuality* indicates how often an event tends to occur. Additional explanation is provided in Table 2. Both these selective resources are embedded in the system network.

Our DNN-based model employs a Bi-LSTM that takes into account the order of tag occurrences. Each input was a sequence of tag sets ***T***^manual^/***T***^auto^. Specifically, for each word, the sum of embedding vectors corresponding to each tag was calculated to obtain the tag embeddings ***E***_tag_∈ℝ^*L*×*d*^, where *d* represents the number of dimensions. These embeddings were then input into the Bi-LSTM. Differentiation was accomplished by inputting the last state of the Bi-LSTM into a fully connected layer. We trained the model for 50 epochs with a batch size of 32, using the Adam optimizer and a learning rate of 0.001. The dimensions of the input word vectors and the hidden layer were 300. These hyperparameters were adopted from commonly used values and not explored, as preliminary experiments determined that the impact of the search for hyperpatameters was minimal. The architecture of the model is depicted in Fig 5.

**(2) Differentiation Using Text**

The model is almost identical to the DNN-based tag model. The only difference is that the input to Bi-LSTM is changed to word embeddings ***E***_word_∈ℝ^*L*×*d*^ instead of tag embeddings ***E***_tag_.

**(3) Differentiation Using Tag-and-Text Combinations**

In tag-based differentiation, the sequence ***T***^manual^ / ***T***^auto^ of tag sets was derived from the input ***x***, leading to a certain degree of information loss. For example, the words *sad* and *angry* both received the same *Attitude-Affect-Emotion tag* (Table 2). By retaining text information, we could differentiate between these words, thereby enhancing differentiation accuracy. To preserve text information, we developed a model that combines text and tags, taking into account complex word-tag relationships while retaining all the related information. For instance, the model can take into account specific situations, such as when a particular word with a specific part of speech appears before or after a certain tag, potentially indicating an AS or non-AS characteristic.

**Fig 6:**
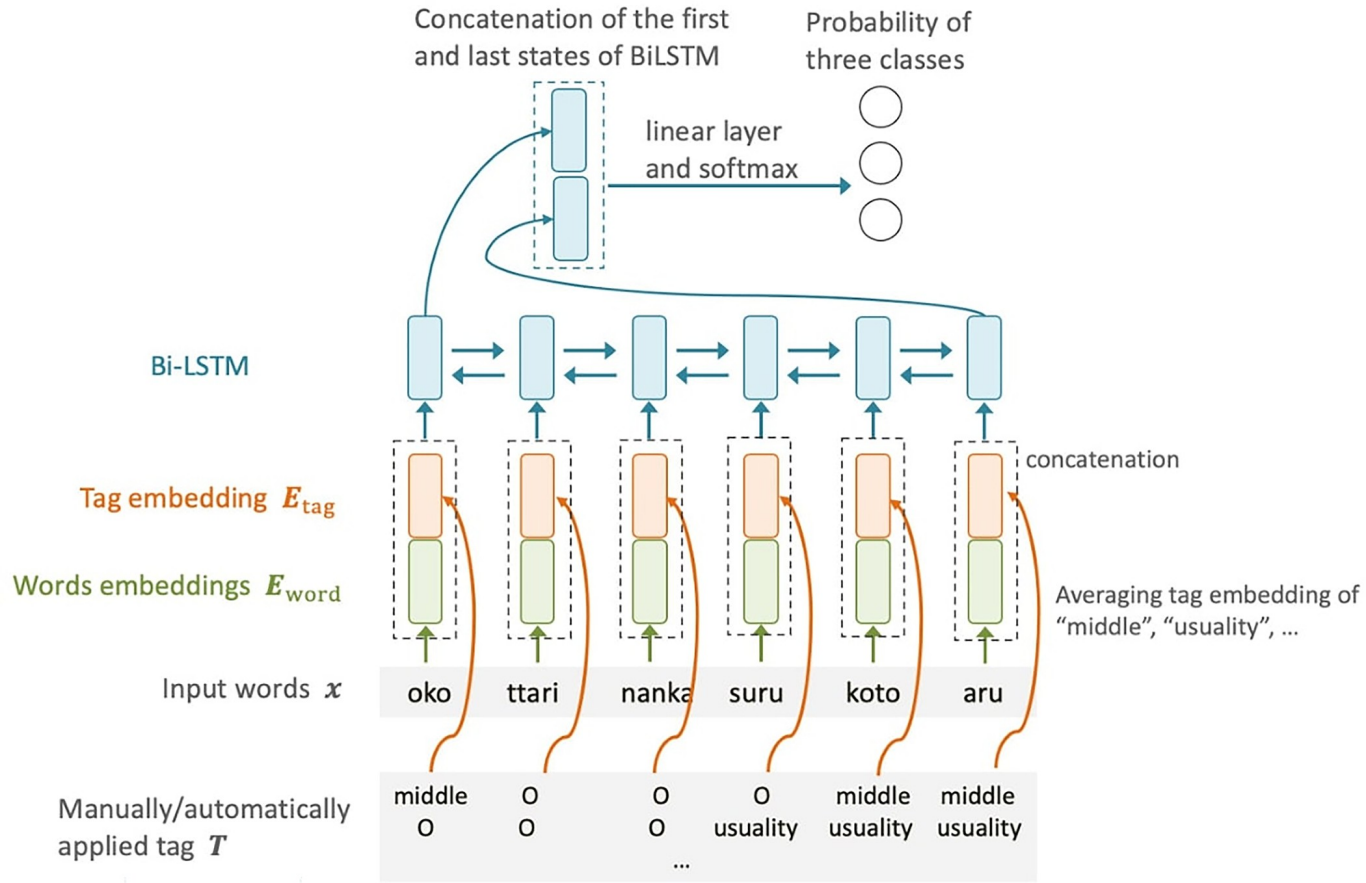
Bi-LSTM-based classification model that utilizes tag-and-text. An example sentence is *Oko ttari nanka surukoto aru* (There are occasions when I get mad). The assigned tags are the same as in Fig 5.

In this model, we employed a Bi-LSTM quite similar to the Bi-LSTM used in the tag-based model. Each input was an ***E***_concat_∈ℝ^*L*×2*d*^, a concatenation of tag embeddings ***E***_tag_ and word embeddings ***E***_word_ at each time step. The methods for predicting ***T***^auto^ and the hyperparameters were identical to those used in the tag-based model. Fig 6 shows the architecture of the model.

## Results

Table 3 presents the accuracies, precisions, sensitivities, and specificities of the tag-linear, tag-DNN, tex-DNN, and text+tag-DNN models, following both automatic and manual annotation. As previously mentioned, manual annotation tends to yield more accurate results, as reflected in the generally higher values compared to automatic annotation. The overall mean F value was 0.88. The complexity of machine annotation, however, has compromised accuracy. For instance, the *te*-clause, one of the annotation categories listed in Table 2, was most frequently associated with errors. This category is subdivided into eight different classifications: conjunctive clause-parallel, conjunctive clause-contrast, conjunctive clause-forerunner, conjunctive clause-sequence of actions, conjunctive clause-cause/reason, conjunctive clause-adversative connective, conjunctive clause-resultative condition, and conjunctive clause-attendant circumstance. Accurate annotation must distinguish these eight types based on morphemes (i.e., *te* and the surrounding words). To improve this, more precise definitions of the differences are necessary, or alternatively, expanding the training data for machine learning could improve the system’s ability to accurately handle the complexities of *te*-clauses.

**Table 3:** Statistical values of linear and DNN-based models.

	Input	Model	Accuracy		95% confidence interval		Precision		Sensitivity		Specificity		AUC	
Annotation Text			manual	automatic	manual	automatic	manual	automatic	manual	automatic	manual	automatic	manual	automatic
Interview	tag	linear model	0.78	0.78	[0.71, 0.85]	[0.71, 0.85]	0.75	0.72	0.77	0.84	0.79	0.73	0.85	0.84
	tag	DNN model	0.80	0.75	[0.73, 0.87]	[0.67, 0.82]	0.77	0.66	0.79	0.93	0.81	0.60	0.88	0.85
	text	DNN model	0.80		[0.73, 0.87]		0.81		0.75		0.85		0.89	
	text + tag	DNN model	0.80	0.82	[0.73, 0.87]	[0.75, 0.89]	0.82	0.80	0.73	0.80	0.87	0.84	0.90	0.90
Picture book recounting	tag	linear model	0.64	0.60	[0.55, 0.73]	[0.51, 0.69]	0.55	0.50	0.52	0.48	0.72	0.69	0.67	0.63
	tag	DNN model	0.75	0.67	[0.67, 0.83]	[0.58, 0.75]	0.69	0.58	0.66	0.57	0.81	0.73	0.81	0.72
	text	DNN model	0.76		[0.68, 0.84]		0.76		0.57		0.88		0.78	
	text + tag	DNN model	0.76	0.78	[0.68, 0.84]	[0.71, 0.86]	0.72	0.78	0.64	0.64	0.84	0.88	0.80	0.80

We used the McNemar test to evaluate whether there are significant differences in accuracy between methods. Specifically, we compared the accuracy between tag-linear model and tag-DNN model, between tag-DNN model and text+tag-DNN model, and between text-DNN model and text+tag-DNN model. For each comparison, the null hypothesis was that there is no significant difference in accuracy between the methods, while the alternative hypothesis was that there is a significant difference in accuracy between the methods.

The results generated by the tag-linear, tag-DNN, and text+tag-DNN models did not display significant differences (Table 4). Nevertheless, the tag-DNN model exhibited a marginal performance improvement over the tag-linear model, and the text+tag-DNN model was slightly superior to the tag-DNN model. The text-DNN and text+tag-DNN models performed almost the same, with the text+tag-DNN model being marginally higher. The absence of statistically significant differences in our McNemar test results does not definitively indicate the absence of a performance difference between models, particularly in the context of small sample sizes. This aligns with broader discussions on statistical power and interpreting non-significant results in research [115–117]. In general, the linear model was less precise than its DNN-based counterparts.

**Table 4:** McNemar test results for model performance comparisons.

		manual		automatic	
		statistic	p-value	statistic	p-value
A	tag(linear model) vs tag (DNN model)	0.04	0.84	0.30	0.58
	tag(DNN model) vs text+tag (DNN model)	0.00	1.00	2.21	0.14
	text(DNN model) vs text+tag (DNN model)	0.12	0.72	0.10	0.75
E	tag(linear model) vs tag (DNN model)	5.50	0.02	1.33	0.25
	tag(DNN model) vs text+tag (DNN model)	0.00	1.00	5.76	0.02
	text(DNN model) vs text+tag (DNN model)	0.12	0.72	0.44	0.50

We acknowledge the limitations of our sample size. Due to constraints, achieving a larger dataset was not feasible. Consequently, our study should be viewed as exploratory, aimed at providing initial insights rather than definitive conclusions.

Regarding the comparison between interview text and story-recounting test, our findings suggest that the interview task may provide insights into the linguistic behaviors of individuals with AS that are more detailed than those provided by the story-recounting task. Given the inherently interactive and social nature of the interview task, it has the potential to highlight differences in lexicogrammatical use that relate to the neurocognitive characteristics of AS. Although the story-recounting task is also social, its monologic nature offers fewer opportunities for such distinctions to emerge. Therefore, in the context of our study, the interview task proved to be a more effective diagnostic tool.

## Discussion

### Implications of the tag-linear model

The working hypothesis of this study suggests that language output may be indicative of underlying cognitive processes. Therefore, we proposed that neurodevelopmental disorders can be distinguished from non-AS conditions through their lexicogrammatical choices. Using the text + tag DNN model and manual annotation, the test displayed results with 80% accuracy, 82% precision, 73% sensitivity, and 87% specificity for the interview texts. These findings indicate the potential of utilizing lexicogrammatical choices as a diagnostic tool, reinforcing our proposition that cognitive patterns influence language output. This notion aligns with the idea that cognitive processes guide lexicogrammatical choices during language formation, as outlined by the SFL stratification in Fig 1 [58].

When devising differentiation criteria, our attention centered on the *lexicogrammar* situated in the fifth stratification layer. To reach lexicogrammar, one must traverse the prior four layers: *cognition*, *culture*, *situation*, and *discourse semantics*. Our system for differentiation rests on the premise that there would be a discernible difference in the lexicogrammatical selections between AS and non-AS individuals within the *lexicogrammar* layer. The articulated lexicogrammar acts as a clear manifestation of the speaker’s chosen syntactic configurations and stands apart due to its sheer objectivity, void of the subjectivity often seen in semantic evaluations. This same principle of objectivity extends to the *phonology/graphology* layer, situated directly below *lexicogrammar*, ensuring the observational standards are purely objective.

Driven by the *register* (located in the third layer), we employ varied expressions. Several factors shape these choices: the context of the conversation (*Field*), the relationship with and societal role of the person we are conversing with (*Tenor*), and the mode of communication (*Mode*), which encompasses aspects like whether the language is spoken or written, the level of formality, and whether the communication is dialogic or monologic. In the act of constructing a clause encapsulating our intended meaning, specific linguistic decisions arise. During speech, speakers instantaneously sift through the system network, effectively engaging in a resource-selection mapping process. This network encapsulates all available lexicogrammatical options during a linguistic exchange. Language functions as a structured system where meanings are crafted by speakers drawing words from a reservoir within the system network, all while partaking in societal activities [112].

### Advantages of the DNN model over the linear model

The superiority of the DNN model can be attributed to its ability to construct judgment criteria through autonomous learning of input data. Deep learning algorithms are proficient at automatically extracting and assimilating the most beneficial features that guarantee output accuracy. This is why the DNN model is a notch above the linear model, given that the DNN incorporates learned judgment criteria alongside the tag information.

The learned criteria function as black boxes, and it is plausible that the DNN considered tag orders and combinations. For instance, the DNN may have identified patterns such as the presence of tag B following tag A indicating autism, the co-occurrence of tags A and B signifying autism, or the independent presence of tag A suggesting non-AS status. In contrast, the linear model’s accuracy is potentially lower than the DNN models’ accuracies due to its constrained input information. This constraint stems from the selection of arbitrary items and the omission of certain data, which restricts the comprehensiveness of the model.

We acknowledge the potential benefits of a text-only model. However, our focus on the tag-only and tag+text models is based on three key reasons:

1. **Medical transparency**: As stated previously, transparency is crucial in medical applications. The lexicogrammatical tags provide clear, interpretable insights, which are essential for effective and transparent use in clinical settings.
2. **Improving Diagnostic Accuracy**: Our study, as a pilot, aimed to demonstrate the potential of these models. While the text+tag approach shows slightly better accuracy than the tag-only approach, we plan to increase the accuracy further by adding more annotation categories from the system network. Currently, we use 147 categories, but expanding this will enhance diagnostic precision. We assume that increasing annotation items from the system network will improve diagnostic accuracy. The text-DNN model has reached its limit in terms of precision, and enhancing accuracy beyond this point will require expanding the system network categories. As mentioned previously, improving diagnostic accuracy is critical, especially for adults with comorbidities where traditional tools struggle.
3. **Cognitive Insights**: The tag-based approach allows us to pinpoint specific lexico-grammatical features linked to neurodevelopmental dysfunctions, aiding in understanding the underlying cognitive processes.

Although the annotation process might seem complex, the text+tag DNN model is efficient due to our developed automatic annotation system. This system streamlines the process by quickly providing classification results upon uploading the transcript and allows for easy verification through downloadable annotated transcripts. We anticipate that the accuracy of the automatic annotation will significantly improve by increasing the amount of training data. Currently, the accuracy of our automatic annotation system is strong, and adding more training data will undoubtedly enhance its precision. The primary problem is that transcription still requires a considerable amount of manual corrections due to the current accuracy limitations of Automatic Speech Recognition (ASR) in Japanese. We acknowledge this as an area for improvement.

### Text appropriate for diagnostic differentiation

We examined interview and story-recounting texts from Modules 3 and 4 of the ADOS-2, discovering that individuals with AS’s lexicogrammatical choices during interviews differed more significantly from those of non-AS individuals compared to story-recounting tasks (Table 3). This observation suggests that, in monological language use, the lexicogrammatical distinctions between AS and non-AS individuals are less marked than in interactive social language situations, highlighting the specific challenges faced by individuals with AS in reciprocal social communication. These results underscore the central issue of social impairment in AS, a neurodevelopmental disorder where difficulties in selecting suitable lexicogrammatical structures for effective interpersonal communication are prominent. Given that social components of language development start forming in early childhood [118], it is expected that children with AS, who have core deficits in social interaction and a limited interest in social engagement, would show significant language development impairments. These social deficits are often linked to cognitive, motor, and sensory challenges, including limited joint attention, weak central coherence, and impaired executive functions.

### Versatility of annotation scheme for our differentiation system

The annotation scheme was based on a Japanese system network constructed specifically for this project using *transfer comparison*. A system network is a language that highlights special features of that language [119]. The description of a particular language without making assumptions based on other languages requires an inordinate amount of time; such a description entails many observations of discursive instances and extensive discourse analysis. Therefore, one practical heuristic method models the description of one language on the descriptions of others. This is *transfer comparison* [119, 120]. Fundamentally, transfer comparison highlights similarities between two languages [120]. We developed the system network of our current annotation scheme using transfer comparison; the descriptive assumptions were based on English because system networks for English are available [113, 121]. Each language is distinct in terms of its descriptors and system network. However, when comprehensive descriptions of some languages are available, typological generalizations across languages become possible. Transfer comparison enables such generalization. Thus, the annotation scheme of Kato et al. [58] is applicable to any language via transfer comparison.

### Limitations and future perspectives

#### Verification process and methodological enhancements

This research constitutes an initial phase in illustrating the feasibility of utilizing a diagnostic instrument for the evaluation of lexicogrammatical choices. The subsequent phase entails a comprehensive verification of this tool: A key limitation of our study is the small sample size. To robustly validate the algorithm developed, expanding the participant pool will be crucial. This will require overcoming logistical challenges and ensuring a larger, more diverse sample to enhance the validity and generalizability of our findings.

Our text+tag DNN model demonstrates efficiency due to the implementation of our automatic annotation system. This system optimizes the process by rapidly providing classification results upon transcript upload and facilitates straightforward verification through downloadable annotated transcripts. We anticipate that increasing the volume of training data will significantly enhance the accuracy of the automatic annotation. Presently, the system exhibits strong accuracy, and expanding the training data set is likely to further refine its precision.

While the text+tag DNN model benefits from our efficient automatic annotation system, the primary challenge remains in the transcription phase. The current limitations of ASR for Japanese necessitate substantial manual corrections. While the text+tag DNN model benefits from our efficient automatic annotation system, the primary challenge remains in the transcription phase. The current limitations of ASR for Japanese necessitate substantial manual corrections. Recognizing this, we have adopted manual transcription for our research to ensure the highest accuracy. However, manual transcription is time-consuming and not feasible for broader clinical applications. Thus, enhancing the ASR system is essential for converting raw voice data into text more efficiently, which is crucial for scaling clinical applications and streamlining the diagnostic process.

#### Analysis of false positive and false negative

A notable limitation of our study is the sensitivity and specificity of the diagnostic tool, both approximately 80%. This suggests a potential 20% error rate in AS diagnosis, manifesting as false negatives or positives. This limitation indicates that in some instances, cases cannot be accurately judged based solely on lexicogrammatical choices. The findings underscore the complexity of diagnosing AS based solely on linguistic patterns, given the broad spectrum and variability in language use within the AS population. This necessitates a more detailed analysis of lexicogrammatical choices and may require adjustments to the annotation scheme, incorporating additional resources from system networks.

To further elucidate, the issue of false negatives and positives can be examined more specifically. In terms of false negatives, this issue may be particularly relevant in individuals with AS characteristics akin to AS individuals without language and cognitive delay, who may exhibit language patterns similar to non-AS individuals. Given that DSM-5 encompasses Asperger’s under the broader AS classification, our study included participants with such complex vocabularies and refined speech, which could lead to diagnostic challenges. Regarding false positives, it is possible that some individuals were misdiagnosed as having AS due to their frequent use of certain lexicogrammatical choices commonly seen in AS, despite being non-AS.

Future research should focus on refining diagnostic criteria and tools to better accommodate the diversity in language use among individuals with AS. Exploring more comprehensive and nuanced methods for differentiating between AS and non-AS individuals, particularly those with atypical language profiles, will be crucial in reducing false diagnostic rates.

#### Investigation of influences of comorbid conditions on lexicogrammatical choices

Our methodology begins with creating a classifier that distinguishes AS from non-AS, a foundational step towards developing a comprehensive diagnostic tool for real-world clinical assessments. We have found discernible differences even without excluding comorbidities, underscoring the potential utility of our research as a diagnostic tool in these complex clinical scenarios. However, further investigation is needed into how comorbidities might affect the occurrence of false positives or negatives. To address this, our next step involves developing separate tools for each comorbid conditions, including, adjustment disorder/non-adjustment disorder, depression/non-depression, ADHD/non-ADHD and so on. This approach aligns with clinical realities and will be crucial in enhancing the accuracy and applicability of our diagnostic tools.

## Conclusions

This study demonstrates the feasibility of using natural language processing (NLP) to develop a diagnostic tool for AS. The text+tag DNN model distinguishes AS from non-AS through lexicogrammatical analysis, indicating significant diagnostic potential. By examining lexicogrammatical choices, our approach shows promise in supporting the multidisciplinary diagnosis of AS. Leveraging NLP and machine learning, we aim to integrate language-based diagnostics with traditional methods, potentially enhancing early detection and support for individuals with AS.

## Supporting information

S1 File Supplementary matessrials. (DOCX)

S2 File Supplementary tables. (DOCX)
