# Correction: Creating a diagnostic assessment model for autism spectrum disorder by differentiating lexicogrammatical choices through machine learning

**DOI:** 10.1371/journal.pone.0320613

**Published:** 2025-04-02

**Authors:** The PLOS One Staff

## Notice of Republication

This article was republished on March 13, 2025, to correct minor typographical errors that were introduced during the typesetting process. The publisher apologizes for these errors. Please download this article again to view the correct version. The originally published, uncorrected article and the republished, corrected articles are provided here for reference.

## Supporting Information

S1 File Originally published, uncorrected article. (PDF)

S2 File Republished, corrected article. (PDF)
