# Supplementary material for: Creating a diagnostic assessment model for autism spectrum disorder by differentiating lexicogrammatical choices through machine learning
Source: PLoS One. 2024 Sep 27;19(9):e0311209. doi: 10.1371/journal.pone.0311209 (PMC11432897; doi:10.1371/journal.pone.0311209)
Supplement: S1 File — (DOCX) [file pone.0311209.s001.docx]

**S1 File. Supplementary Material.**

**S1 Material 1.** **Interview questions from Module 4 [1].**

**Social Difficulties and Annoyance**

**FOR ADULTS (PRESENTLY NOT IN SCHOOL)**

- Do you have a job?

***If so:***

• What kind of job is it? How did you find it?

• Have you had other jobs before?

• Are you happy where you are or would you like to move on to something else eventually? What would it be?

• What about your co-workers? Do they seem to be happy or are they ready to move on?

***If not:***

• What do you do during the day?

• Did you have a job before? Why did you leave your old job? Was it something you had planned?

• Would you like to have a job someday?

***If yes:***

- What would it be?

- What will you need to do to find this type of job?

***If no:***

- What would you like to do?

**OR FOR STUDENTS (ABOVE JUNIOR HIGH SCHOOL AGES)**

Interview Questions About School

- Are you in school? Where?
- What courses are you taking?
- What year (grade) are you in? How is it going?

***If the participant is no longer in school and not employed:***

- How far did you go in school? How did it go?
- What do you plan to do next? What experience/training would you need to do that?
- Have you ever saved your money to buy something or do something special? What was it?
- Where are you living now?

***If living at home with parents:***

• Have you ever lived away from your parents?

• What would be different about living on your own?

• Would you like it better?

• What would be difficult?

***If living on his or her own:***

• How did you find the place where you live now?

• Who do you live with?

• Can you tell me a little about it?

- What do you like to do in your spare time at home?
- What about going out?
- What do you like doing that makes you feel happy and cheerful?
- What about things that you’re afraid of? What makes you feel frightened or anxious? How does it feel? What do you do?
- What about feeling angry? What kinds of things make you feel that way? How do you feel “inside” when you’re angry?
- Most people have times when they feel sad. What kinds of things make you feel that way?
- How do you feel when you’re sad? What is it like when you’re sad? Can you describe that?
- How about feeling relaxed or content? What kinds of things make you feel that way?
- Have you ever had problems getting along with people at school? How about at home with your family? Do you ever get in trouble? Why? What for?
- Are there things that other people do that irritate or annoy you? What are they?
- What about things you do that annoy others?-(if no response, ask: What about your brother(s) or sister(s) or parents?)
- Have you ever been teased or bullied? Why, do you think?
- Have you ever tried to change these things? Have you ever done anything so that others wouldn’t tease you? How has it worked?
- Are there other kids/people you know who get teased or bullied?
- Do you have some friends? Can you tell me about them?
- What do you like doing together? How did you get to know them? How often do you get together?
- What does being a friend mean to you? How do you know someone is your friend?
- How is a friend different from someone whom you just go to school with?
- Do you have a girlfriend or boyfriend? What is her/his name? How old is she/he?
- When did you see her/him last?
- What is she/he like? What do you like to do together?
- How do you know she/he is your girlfriend/boyfriend?
- Where do you want to live when you get older? What kind of place (apartment, house, condo)?
- Whom do you think you would like to live with? Your family, a roommate(s), by yourself?
- Do you ever think about having a long-term relationship or getting married (when you are older)?
- Why do you think some people get married or live with a girlfriend or boyfriend when they grow up?
- What would be nice about it? What might be difficult about being married or living with a girlfriend or boyfriend? Or living with a roommate?
- Do you ever feel lonely?
- Do you think other kids/people your age ever feel lonely?
- Are there things that you do to help yourself feel better? What about things other people do to help themselves feel better when they’re lonely?

***S1 Material 2. System networks.***

**S1 Fig 1. The system network of MOOD (mood selection) in Japanese [2].** This was modified from Teruya [3] and Kadooka et al. [4]. MOOD focuses on the interactional meaning, viewing the clause as a verbal exchange between interlocutors. The red-circle part is in Figure 4. The speakers are expected to choose one from a set of oppositions, with the degree of delicacy increasing from left to right on the network. The current annotation scheme incorporated the items in the green-colored portions.


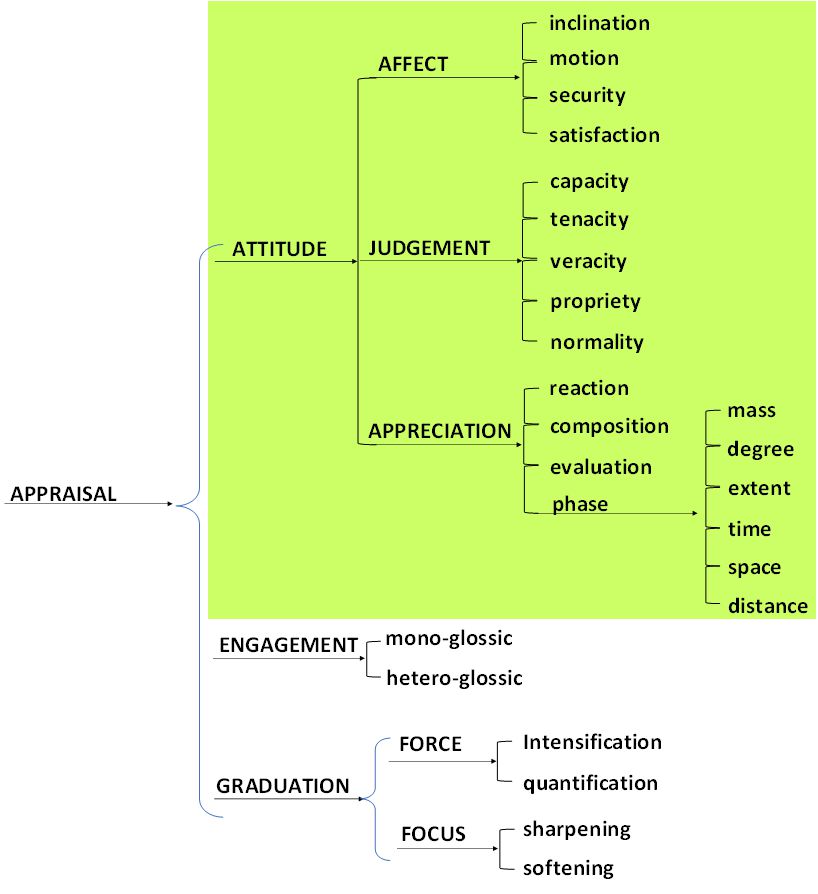


**S1 Fig 2. System network of APPRAISAL in Japanese [2]**. This was constructed by transfer comparison following Martin and White [5]. APPRAISAL handles the evaluative meanings constructed by speakers. The current annotation scheme incorporated the items in the green-colored portions.


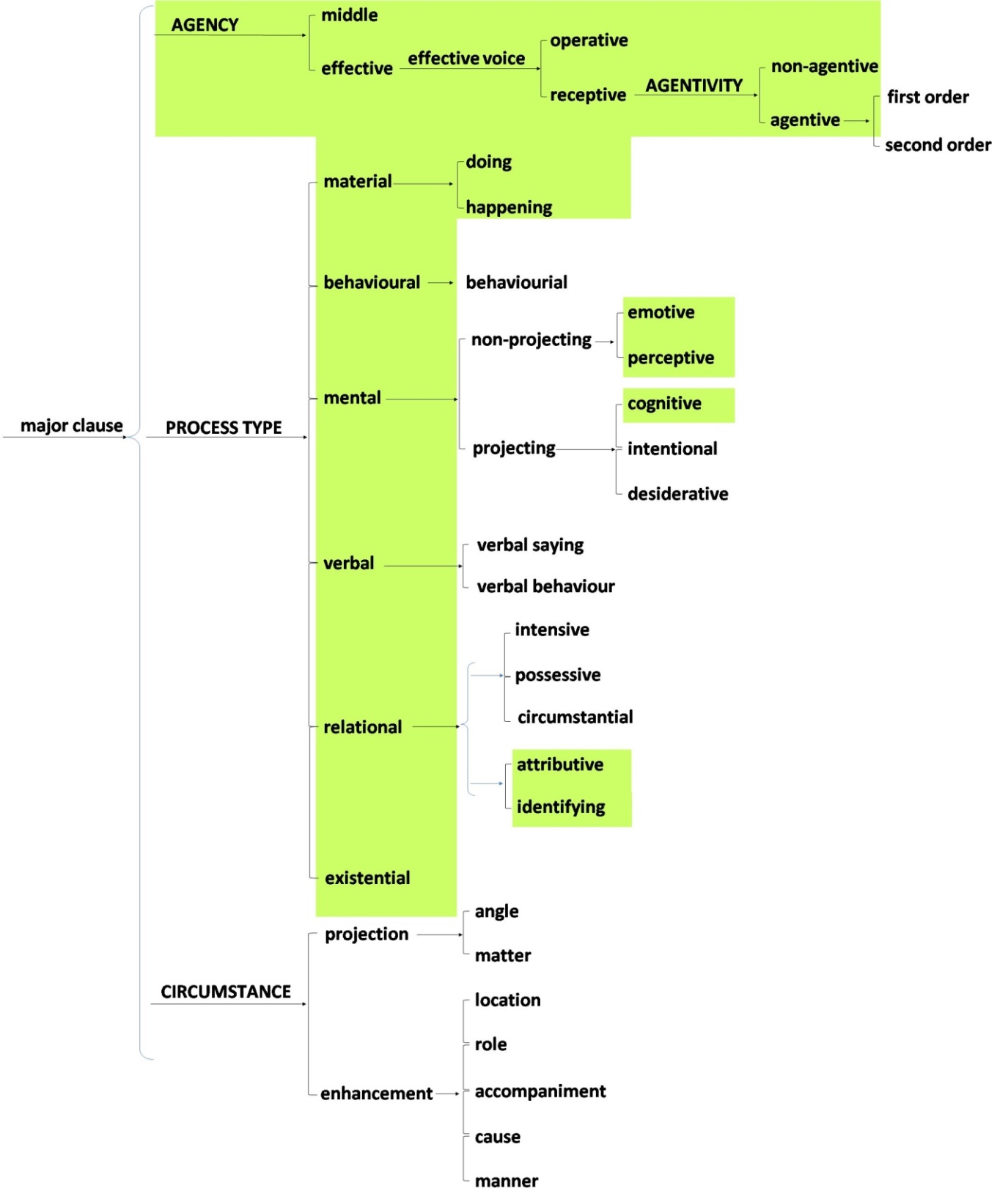


**S1 Fig 3. System network of TRANSITIVITY in Japanese [2].** This was constructed by transfer comparison following Matthiessen [6]. TRANSITIVITY deals with components of the clause. The current annotation scheme incorporated the items in the green-colored portions.


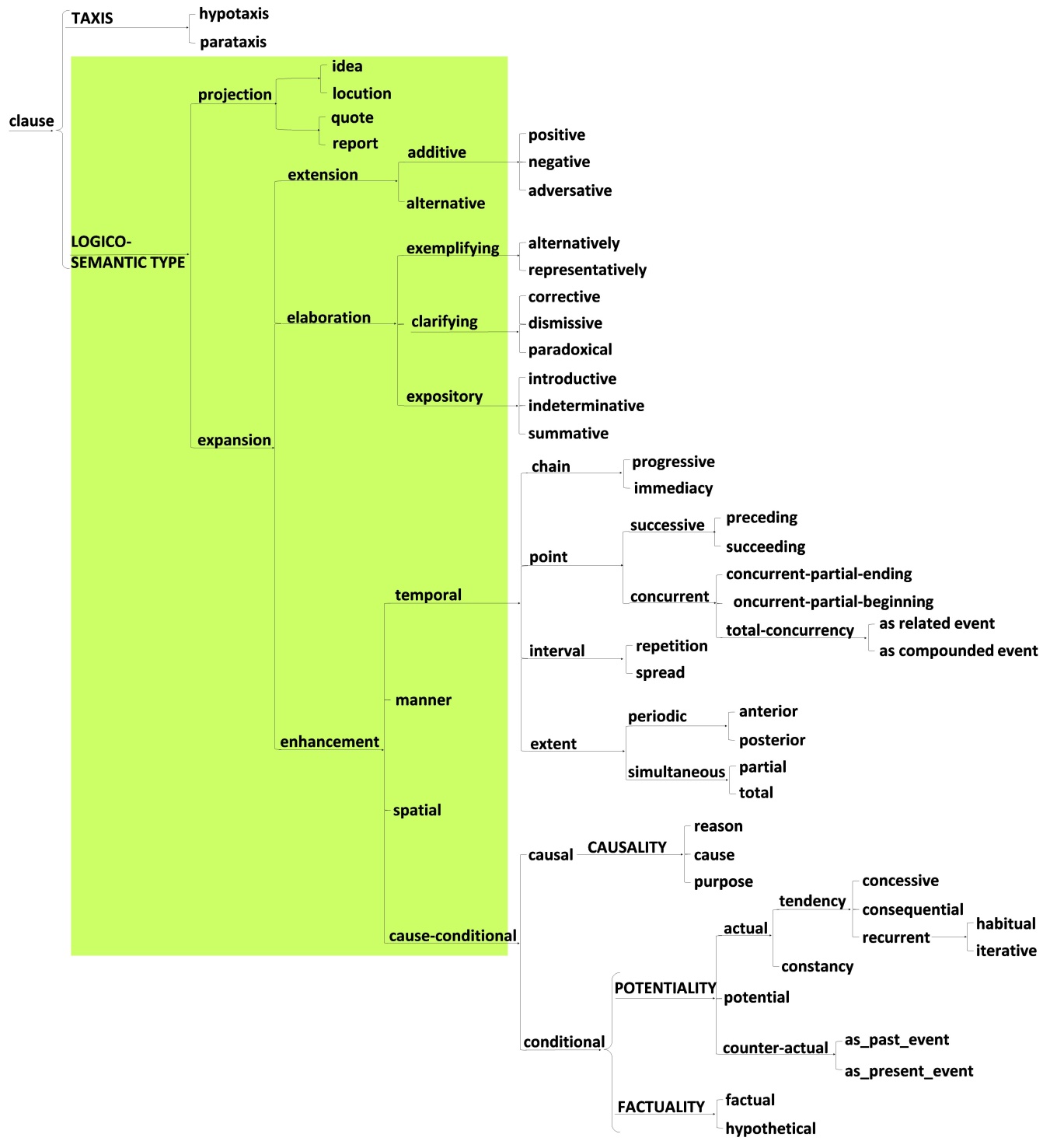


**S1 Fig 4. LOGICAL systems in Japanese [2]**. This was constructed by transfer comparison following Halliday and Matthiessen [7]. LOGICAL manages relationships between clauses within the clause complex. The current annotation scheme incorporated the items in the green-colored portions.

**References**

1. Corsello C, Spence S, Lord C. Autism Diagnostic Observation Schedule, 2nd ed. (ADOS-2) Training Videos Guidebook (Part I): Modules 1- 4. Torrance, CA: Western Psychological Services; 2012.
2. Kato S, Hanawa K, Linh VP, Saito M, Iimura R, Inui K, et al. Toward mapping pragmatic impairment of autism spectrum disorder individuals through the development of a corpus of spoken Japanese. PLOS ONE. 2022;17(2): e0264204. doi:10.1371/journal.pone.0264204
3. Teruya K. A systemic functional grammar of Japanese. N.Y.: Continuum; 2007.
4. Kadooka K, Igarashi K, Iimura R, Fukuda K, Kato S. Kino bunpo ni yoru nihongo modariti kenkyu [Studies in Japanese modality based on functional grammar]. Tokyo: Kuroshio; 2016.
5. Martin JR, White PRR. The language of evaluation: Appraisal in English. Palgrave Macmillan; 2005.
6. Matthiessen D. Lexicogrammatical cartography. Tokyo: International Language Science Publisher; 1995.
7. Halliday MAK, Matthiessen C. An introduction to functional grammar, 2nd ed. London: Arnold; 2007.
