# Supplementary material for: Creating a diagnostic assessment model for autism spectrum disorder by differentiating lexicogrammatical choices through machine learning
Source: PLoS One. 2024 Sep 27;19(9):e0311209. doi: 10.1371/journal.pone.0311209 (PMC11432897; doi:10.1371/journal.pone.0311209)
Supplement: S2 File — (DOCX) [file pone.0311209.s002.docx]

**S2 File. Supplementary Tables.**

**S2 Table 1. Normalized Statistical Differences in Lexicogrammatical Item Selection Between AS and Non-AS Groups in interview texts.**

|  | AS | | | |  | non-AS | | | | t-value | p-value |
| --- | --- | --- | --- | --- | --- | --- | --- | --- | --- | --- | --- |
|  | Mean | SD | Min. | Max. |  | Mean | SD | Min. | Max. |  |  |
| Auxiliary Verbs/Benefactive-do~for someone | 0.0000 | 0.0001 | 0.0000 | 0.0004 |  | 0.0002 | 0.0004 | 0.0000 | 0.0029 | 2.940 | 0.004 |
| Auxiliary Verbs/Stative-try doing/to do something and see what happens | 0.0003 | 0.0005 | 0.0000 | 0.0023 |  | 0.0006 | 0.0006 | 0.0000 | 0.0025 | 2.232 | 0.027 |
| Clause complexes/Noun clause | 0.0062 | 0.0035 | 0.0000 | 0.0175 |  | 0.0077 | 0.0038 | 0.0000 | 0.0217 | 2.372 | 0.019 |
| Clause complexes/Reported clause | 0.0104 | 0.0042 | 0.0012 | 0.0210 |  | 0.0126 | 0.0040 | 0.0046 | 0.0282 | 3.197 | 0.002 |
| Clause complexes/Adnominal clause | 0.0082 | 0.0035 | 0.0017 | 0.0175 |  | 0.0098 | 0.0029 | 0.0046 | 0.0156 | 2.986 | 0.003 |
| Clause complexes/*Te*-form/Conjunctive clause-parallel/contrast | 0.0012 | 0.0011 | 0.0000 | 0.0044 |  | 0.0019 | 0.0013 | 0.0000 | 0.0055 | 3.531 | 0.001 |
| Clause complexes/*Te*-form/Conjunctive clause-forerunner | 0.0004 | 0.0005 | 0.0000 | 0.0017 |  | 0.0006 | 0.0006 | 0.0000 | 0.0025 | 2.314 | 0.022 |
| Clause complexes/*Te*-form/Conjunctive clause-cause/reason | 0.0022 | 0.0019 | 0.0000 | 0.0086 |  | 0.0033 | 0.0021 | 0.0000 | 0.0089 | 3.365 | 0.001 |
| Clause complexes/*Te*-form/Conjunctive clause-attendant circumstance | 0.0016 | 0.0014 | 0.0000 | 0.0070 |  | 0.0022 | 0.0014 | 0.0000 | 0.0073 | 2.602 | 0.010 |
| Clause complexes/*Te*-form/Conjunctive clause-sequence of actions | 0.0015 | 0.0016 | 0.0000 | 0.0057 |  | 0.0024 | 0.0015 | 0.0000 | 0.0073 | 3.228 | 0.002 |
| Clause complexes/Parallel clause | 0.0061 | 0.0033 | 0.0000 | 0.0135 |  | 0.0039 | 0.0031 | 0.0000 | 0.0161 | -4.041 | 0.000 |
| Clause complexes/Conditional clause-cause/reason | 0.0037 | 0.0025 | 0.0000 | 0.0110 |  | 0.0049 | 0.0028 | 0.0000 | 0.0166 | 2.704 | 0.008 |
| Clause complexes/Conditional clause-converse condition-adversative connective | 0.0006 | 0.0006 | 0.0000 | 0.0031 |  | 0.0008 | 0.0008 | 0.0000 | 0.0033 | 2.150 | 0.033 |
| Clause complexes/Conditional clause-resultative condition | 0.0036 | 0.0021 | 0.0000 | 0.0080 |  | 0.0043 | 0.0020 | 0.0007 | 0.0082 | 2.055 | 0.042 |
| Logico-Semantic Relation/Projection-embedding | 0.0148 | 0.0051 | 0.0017 | 0.0238 |  | 0.0181 | 0.0055 | 0.0089 | 0.0352 | 3.541 | 0.001 |
| Logico-Semantic Relation/Projection-idea | 0.0074 | 0.0037 | 0.0009 | 0.0177 |  | 0.0094 | 0.0035 | 0.0028 | 0.0245 | 3.186 | 0.002 |
| Logico-Semantic Relation/Expansion-enhancement-manner | 0.0019 | 0.0015 | 0.0000 | 0.0079 |  | 0.0026 | 0.0015 | 0.0000 | 0.0073 | 2.574 | 0.011 |
| Logico-Semantic Relation/Expansion-enhancement-cause-conditional | 0.0100 | 0.0050 | 0.0000 | 0.0215 |  | 0.0132 | 0.0040 | 0.0035 | 0.0243 | 4.103 | 0.000 |
| Logico-Semantic Relation/Expansion-extension-additive | 0.0082 | 0.0044 | 0.0000 | 0.0200 |  | 0.0111 | 0.0033 | 0.0062 | 0.0208 | 4.175 | 0.000 |
| Logico-Semantic Relation/Expansion-elaboration-exemplifying | 0.0059 | 0.0033 | 0.0000 | 0.0135 |  | 0.0036 | 0.0031 | 0.0000 | 0.0154 | -4.150 | 0.000 |
| Process Type/Existential | 0.0157 | 0.0063 | 0.0044 | 0.0320 |  | 0.0114 | 0.0037 | 0.0046 | 0.0252 | -4.731 | 0.000 |
| Process Type/Relational-attribute | 0.0212 | 0.0064 | 0.0082 | 0.0408 |  | 0.0241 | 0.0050 | 0.0116 | 0.0409 | 2.885 | 0.005 |
| Appraisal/Attitude/JUDGEMENT-propriety | 0.0006 | 0.0007 | 0.0000 | 0.0031 |  | 0.0009 | 0.0008 | 0.0000 | 0.0039 | 2.560 | 0.012 |
| Appraisal/Attitude/JUDGEMENT-veracity | 0.0005 | 0.0006 | 0.0000 | 0.0027 |  | 0.0009 | 0.0012 | 0.0000 | 0.0050 | 2.325 | 0.022 |
| Appraisal/Attitude/AFFECT-satisfaction | 0.0004 | 0.0005 | 0.0000 | 0.0025 |  | 0.0014 | 0.0012 | 0.0000 | 0.0059 | 6.350 | 0.000 |
| Appraisal/Attitude/APPRECIATION-reaction | 0.0093 | 0.0040 | 0.0028 | 0.0189 |  | 0.0106 | 0.0035 | 0.0036 | 0.0231 | 2.054 | 0.042 |
| Appraisal/Graduation/FORCE-intensification | 0.0037 | 0.0025 | 0.0000 | 0.0098 |  | 0.0085 | 0.0049 | 0.0000 | 0.0246 | 7.218 | 0.000 |
| Appraisal/Graduation/FORCE-quantification | 0.0011 | 0.0010 | 0.0000 | 0.0051 |  | 0.0015 | 0.0011 | 0.0000 | 0.0046 | 2.398 | 0.018 |
| Evidentiality/Appearance | 0.0006 | 0.0007 | 0.0000 | 0.0029 |  | 0.0009 | 0.0009 | 0.0000 | 0.0053 | 2.222 | 0.028 |
| Modality/Modalization /Probability | 0.0009 | 0.0011 | 0.0000 | 0.0042 |  | 0.0015 | 0.0013 | 0.0000 | 0.0062 | 3.005 | 0.003 |
| Modality/Modulation/Obligation | 0.0000 | 0.0002 | 0.0000 | 0.0012 |  | 0.0002 | 0.0003 | 0.0000 | 0.0015 | 2.920 | 0.004 |
| Negotiator particle/sentence-ending particles *kane* | 0.0009 | 0.0020 | 0.0000 | 0.0105 |  | 0.0018 | 0.0022 | 0.0000 | 0.0130 | 2.619 | 0.010 |
| Negotiator particle/sentence-ending particles *ne* | 0.0035 | 0.0044 | 0.0000 | 0.0204 |  | 0.0071 | 0.0062 | 0.0000 | 0.0230 | 3.918 | 0.000 |
| Negotiator particle/sentence-ending particles *yo* | 0.0006 | 0.0010 | 0.0000 | 0.0053 |  | 0.0017 | 0.0022 | 0.0000 | 0.0090 | 4.033 | 0.000 |
| Negotiator particle/sentence-ending particles *yone* | 0.0002 | 0.0005 | 0.0000 | 0.0026 |  | 0.0007 | 0.0010 | 0.0000 | 0.0042 | 3.608 | 0.000 |
| Negotiator particle/Particle *kane* | 0.0000 | 0.0000 | 0.0000 | 0.0000 |  | 0.0001 | 0.0002 | 0.0000 | 0.0016 | 2.642 | 0.010 |
| Negotiator particle/Particle *ne* | 0.0004 | 0.0011 | 0.0000 | 0.0061 |  | 0.0015 | 0.0025 | 0.0000 | 0.0143 | 3.574 | 0.001 |
| Mood/Explanative mood-*kedo* | 0.0030 | 0.0026 | 0.0000 | 0.0123 |  | 0.0039 | 0.0025 | 0.0000 | 0.0100 | 2.086 | 0.039 |
| Mood/Explanative mood-*ne* | 0.0000 | 0.0000 | 0.0000 | 0.0002 |  | 0.0001 | 0.0003 | 0.0000 | 0.0018 | 2.701 | 0.009 |
| Mood/Explanative mood-*yo* | 0.0004 | 0.0008 | 0.0000 | 0.0049 |  | 0.0012 | 0.0016 | 0.0000 | 0.0070 | 3.922 | 0.000 |
| Mood/Explanative mood-*yone* | 0.0001 | 0.0003 | 0.0000 | 0.0017 |  | 0.0003 | 0.0005 | 0.0000 | 0.0027 | 2.981 | 0.003 |
| Filler/*unto* | 0.0022 | 0.0034 | 0.0000 | 0.0159 |  | 0.0010 | 0.0015 | 0.0000 | 0.0076 | -2.648 | 0.010 |
| Filler/*kono* | 0.0000 | 0.0000 | 0.0000 | 0.0003 |  | 0.0001 | 0.0001 | 0.0000 | 0.0007 | 3.047 | 0.003 |

**S2 Table 2. Normalized Statistical Differences in Lexicogrammatical Item Selection Between AS and Non-AS Groups in narrative-recounting texts.**

|  | AS | | | |  | non-AS | | | | t-value | p-value |
| --- | --- | --- | --- | --- | --- | --- | --- | --- | --- | --- | --- |
|  | Mean | SD | Min. | Max. |  | Mean | SD | Min. | Max. |  |  |
| Ergativity/Effective | 0.0393 | 0.0159 | 0.0183 | 0.0786 |  | 0.0323 | 0.0142 | 0.0000 | 0.0598 | -2.427 | 0.017 |
| Auxiliary Verbs/stative-manner at time of movement/to;into/doing something and then going/will continue/disappearance of something or becoming more distant from the speaker | 0.0027 | 0.0031 | 0.0000 | 0.0122 |  | 0.0042 | 0.0040 | 0.0000 | 0.0163 | 2.310 | 0.023 |
| Clause complexes/Cordinate clause | 0.0009 | 0.0016 | 0.0000 | 0.0059 |  | 0.0019 | 0.0027 | 0.0000 | 0.0122 | 2.335 | 0.021 |
| Clause complexes/Noun clause | 0.0018 | 0.0024 | 0.0000 | 0.0112 |  | 0.0029 | 0.0028 | 0.0000 | 0.0123 | 2.244 | 0.027 |
| Clause complexes/Time clause-simultaneous actions | 0.0002 | 0.0007 | 0.0000 | 0.0042 |  | 0.0010 | 0.0018 | 0.0000 | 0.0089 | 3.089 | 0.003 |
| Clause complexes/Conditional clause-converse condition-adversative connective | 0.0005 | 0.0010 | 0.0000 | 0.0045 |  | 0.0009 | 0.0013 | 0.0000 | 0.0052 | 2.077 | 0.040 |
| Clause complexes/Expansion-enhancement-temporal | 0.0015 | 0.0020 | 0.0000 | 0.0070 |  | 0.0023 | 0.0024 | 0.0000 | 0.0115 | 2.138 | 0.035 |
| Logico-Semantic Relation/Expansion-enhancement-cause-conditional | 0.0112 | 0.0081 | 0.0000 | 0.0349 |  | 0.0147 | 0.0079 | 0.0000 | 0.0409 | 2.320 | 0.022 |
| Process Type/Mental-perception | 0.0017 | 0.0024 | 0.0000 | 0.0134 |  | 0.0028 | 0.0030 | 0.0000 | 0.0116 | 2.125 | 0.036 |
| Process Type/Material-doing | 0.0417 | 0.0145 | 0.0163 | 0.0786 |  | 0.0346 | 0.0153 | 0.0000 | 0.0688 | -2.529 | 0.013 |
| Appraisal/Attitude/AFFECT-satisfaction | 0.0001 | 0.0003 | 0.0000 | 0.0016 |  | 0.0005 | 0.0011 | 0.0000 | 0.0070 | 3.154 | 0.002 |
| Appraisal/Attitude/APPRECIATION-phase-time | 0.0010 | 0.0016 | 0.0000 | 0.0057 |  | 0.0017 | 0.0021 | 0.0000 | 0.0075 | 2.062 | 0.041 |
| Appraisal/Attitude/APPRECIATION-phase-mass | 0.0017 | 0.0019 | 0.0000 | 0.0071 |  | 0.0031 | 0.0031 | 0.0000 | 0.0131 | 2.936 | 0.004 |
| Appraisal/Graduation/FORCE-intensification | 0.0011 | 0.0023 | 0.0000 | 0.0117 |  | 0.0040 | 0.0041 | 0.0000 | 0.0210 | 4.857 | 0.000 |
| Appraisal/Graduation/FOCUS-softening | 0.0012 | 0.0021 | 0.0000 | 0.0086 |  | 0.0021 | 0.0027 | 0.0000 | 0.0105 | 1.998 | 0.048 |
| Modality/Modulation/Necessity | 0.0000 | 0.0002 | 0.0000 | 0.0013 |  | 0.0002 | 0.0006 | 0.0000 | 0.0029 | 2.515 | 0.014 |
| Modality/Modulation/Inclination | 0.0000 | 0.0002 | 0.0000 | 0.0014 |  | 0.0004 | 0.0011 | 0.0000 | 0.0062 | 2.718 | 0.008 |
